# Progress in Optical Methods for the Detection of Two Core Blood Biomarkers of Alzheimer’s Disease: Amyloid-Beta and Tau Proteins

**DOI:** 10.3390/bios16080427

**Published:** 2026-08-06

**Authors:** Ning Xia, Fengli Gao, Chuye Zheng

**Affiliations:** Henan Province of Key Laboratory of New Optoelectronic Functional Materials, College of Chemistry and Chemical Engineering, Anyang Normal University, Anyang 455000, China; flgao@aynu.edu.cn (F.G.); 13850182553@163.com (C.Z.)

**Keywords:** amyloid-beta, Tau, colorimetry, fluorescence, chemiluminescence, surface plasmon resonance, surface-enhanced Raman scattering

## Abstract

Alzheimer’s disease (AD) is the most common neurodegenerative disorder worldwide. Early diagnosis of AD is crucial for delaying disease progression and improving patients’ quality of life. Blood biomarkers, particularly amyloid-beta (Aβ) and Tau proteins along with their phosphorylated isoforms, show advantages such as convenient sampling, minimal invasiveness, and excellent repeatability. However, the extremely low concentrations of AD biomarkers in blood impose stringent requirements on the sensitivity, specificity, and anti-interference capability of detection methods. Optical methods provide promising analytical platforms to address these challenges in view of their intrinsic merits of high sensitivity and selectivity; rapid response; and potential for miniaturization. This review systematically summarizes the latest advances in optical methods for the detection of the two core AD blood biomarkers (Aβ and Tau), covering techniques such as colorimetry, fluorescence, chemiluminescence, surface plasmon resonance (SPR), and surface-enhanced Raman scattering (SERS). The sensing principles, design strategies, and analytical performances of these methods are discussed, with special emphasis on different signal amplification strategies. In addition, several challenges and future prospects are provided with a primary focus on single-molecule detection, insufficient sensitivity and stability, lack of validation with large clinical cohorts, and absence of standardization. This review aims to provide researchers with guidance for the rational development of high-performance optical methods to achieve early diagnosis of AD.

## 1. Introduction

As the primary cause of dementia, Alzheimer’s disease (AD) accounts for 60–70% of all dementia diagnoses and has become the seventh leading cause of death worldwide. Over 55 million people globally suffer from Alzheimer’s disease and other dementias, with this number projected to double every two decades [1]. The total cost of associated healthcare has exceeded USD 1.3 trillion and is projected to reach USD 2.8 trillion within ten years. The pathological changes in AD usually begin 15 to 20 years before the first clinical symptoms appear. The hallmark pathological features of AD are senile plaques formed by the aggregation of amyloid-beta (Aβ) proteins and neurofibrillary tangles formed by the deposition of hyperphosphorylated Tau (p-Tau) proteins [2]. During the progression of AD, patients usually exhibit symptoms at the early stage around the age of 65, characterized by progressive declines in language, perception, motor, and executive functions, accompanied by severe impairment of short-term memory. As the disease progresses to the middle and late stages, patients gradually lose the ability to read, write, communicate, and live independently, eventually requiring full-time care. Death often results from complications such as infection, pneumonia, or cardiac arrest. The current diagnostic system for AD is facing severe challenges. Before meeting clinical diagnostic criteria, patients often experience a mild cognitive impairment stage, characterized primarily by reduced abstract thinking and memory loss. Since these symptoms are easily mistaken for normal aging or stress, many mild cognitive impairment patients will eventually be diagnosed with AD, during which neuronal damage is mostly irreversible. Neuroimaging techniques currently used in clinical practice, such as magnetic resonance imaging, computed tomography, and positron emission tomography, can provide important information on brain structure and pathology. However, they are expensive and complex to operate, require specialized facilities, and have radiation risks, making them unsuitable for large-scale screening and long-term monitoring of neurodegenerative diseases [3]. The detection of biomarkers in cerebrospinal fluid (CSF) has high accuracy but relies on lumbar puncture. Such an invasive surgery with low patient acceptance limits its application in early screening in asymptomatic populations [4]. In addition, positron emission tomography and advanced imaging technologies require specialized equipment such as a cyclotron, further limiting their application in primary healthcare settings. Therefore, developing early AD diagnostic techniques that can achieve minimally invasive sampling, rapid detection, low cost, high sensitivity, and high specificity has become a core scientific problem in precision medicine for neurological diseases.

The pathogenesis of AD is remarkably intricate, prominently characterized by the misfolding and aberrant phosphorylation of proteins, sustained neuroinflammation, synaptic loss, and neuronal apoptosis [5]. Among them, the abnormal aggregation of Aβ and Tau proteins is considered the core mechanism driving the occurrence and progression of AD [6]. The concentrations of Aβ and Tau proteins, as two core biomarkers of AD, vary drastically across different biological fluids, posing significant challenges to detection technology [7,8,9]. Conventional methods such as enzyme-linked immunosorbent assay (ELISA) lack sensitivity to quantify ultra-low abundance biomarkers in peripheral blood, and the results from different kits require standardization [10,11,12]. Liquid chromatography-tandem mass spectrometry (LC-MS/MS) offers high sensitivity, but the operation is complex and accuracy as well as excellent multiplexing capability, but the operation is complex and requires specialized personnel, and the sample pre-processing is time-consuming, making it unsuitable for large-scale screening of neurodegenerative diseases. Optical methods with high sensitivity and specificity provide a viable technological pathway to overcome these bottlenecks [13,14]. They can distinguish Aβ monomers from oligomers, identify specific phosphorylation sites on Tau protein, and perform simultaneous quantitative analysis of multiple indicators. In recent years, sensor substrates based on noble metals (gold and silver) and semiconductor materials have been developed, improving detection performance through the synergistic effect of electromagnetic and chemical enhancement [15,16,17]. Researchers have also developed plasmonic nanostructures for specific identification and detection of Aβ and Tau proteins with low detection limits [18,19]. Optical immunosensors can simultaneously detect multiple targets and isoforms (e.g., Aβ40/Aβ42, total Tau, and p-Tau), enabling reliable discrimination between AD patients and healthy controls directly in clinical biofluid samples.

It should be noted that the concentrations of AD biomarkers in blood are extremely low (pg/mL or even fg/mL levels), and there is significant overlap in the distribution of different biomarkers, making them susceptible to biological factors such as age and renal function. Therefore, solely pursuing lower detection limits is insufficient for clinical decision-making, and standardized large-scale population databases must be established to enable dynamic longitudinal tracking. Colorimetric methods offer advantages such as simple operation, independence of sophisticated instruments, and cost-effectiveness, enabling rapid visual detection. Such methods are suitable for preliminary screening of high-risk AD populations in primary care and community settings. Lateral flow assay (LFA) strips based on gold nanoparticles (AuNPs) are representative examples, and their sensitivity can be further improved when combined with magnetic enrichment and machine learning optimization. However, their sensitivity is relatively low (typically at the pM to nM level), making them difficult to meet the requirements for precise quantification of ultra-low-abundance biomarkers. Therefore, colorimetric methods can be employed for preliminary triage screening in different populations, rather than independent confirmatory diagnostic tools. Fluorescence methods offer high sensitivity (up to the fg/mL level) and rapid response, whose performance can be further enhanced by various signal amplification strategies such as fluorescence resonance energy transfer (FRET), clustered regularly interspaced short palindromic repeats (CRISPR)-associated protein (Cas) (CRISPR-Cas) system, and nucleic acid amplification. They can realize accurate quantification of core AD blood biomarkers to support clinical diagnosis and severity assessment. However, the relatively complex operation and high equipment cost limit their applicability for large-scale population screening, and they are primarily suitable for clinical scenarios that require precise quantification. Chemiluminescence assays offer a pg/mL level of sensitivity with high automation, making them suitable for the detection of relatively abundant blood AD protein biomarkers. However, in practical applications, it is necessary to evaluate in advance whether the assay can cover the biomarker concentration ranges across different AD stages to ensure the reliability of detection results. Surface plasmon resonance (SPR) is a label-free real-time detection technique that can monitor the interactions between biomolecules. Its clinical positioning is to conduct confirmatory diagnosis and treatment monitoring in large medical centers, achieving label-free real-time quantitative detection of biomarkers to support dynamic monitoring of AD diagnosis and disease progression. Miniaturized local surface plasmon resonance (LSPR) chips and fiber-optic sensors offer potential for portable detection. However, SPR and LSPR instruments are expensive and have limited throughput, and their ability to detect biomarker concentration changes across different AD stages should be thoroughly evaluated. Surface-enhanced Raman scattering (SERS) methods combine ultra-high sensitivity (down to fg/mL or even ag/mL level) with “molecular fingerprint” specificity, achieving single-molecule level detection through electromagnetic field enhancement and supporting simultaneous multi-target detection in a single test. However, SERS instruments are costly and relatively complex to operate, and spectral reproducibility is affected by batch-to-batch variation in substrates. At present, SERS is primarily indicated for confirmatory diagnosis and clinical trial participant screening in large medical centers, while its application in large-scale screening is still limited by standardization challenges.

Different optical methods have their respective applicable scenarios and limitations within the clinical pathway. The development of effective methods should be matched to practical application needs. With this understanding, this article focuses on the latest advances in optical sensing technologies for the quantitative analysis of Aβ and Tau, two principal blood biomarkers of AD. We systematically summarize the sensing principles, design strategies, and analytical performances of five major optical biosensing techniques, including colorimetry, fluorescence, chemiluminescence, SPR, and SERS. Special emphasis is placed on different signal readout and amplification strategies, such as enzyme/nanozyme-catalyzed chromogenic reactions, aggregation-induced color changes, nucleic acid-based signal amplification, antibody/aptamer-based label-free or sandwich assays, and artificial intelligence (AI)-assisted analysis. In addition, emerging directions including multi-biomarker detection, microfluidic integration, and single-molecule immunoassay are discussed, along with the introduction of current challenges such as insufficient sensitivity, lack of clinical validation, and absence of standardization. This review aims to provide a systematic reference for the rational design of high-performance optical methods and promote the translation of biomarker detection from laboratory research to clinical practice.

## 2. Aβ and Tau Proteins

Aβ is a peptide or protein of approximately 4 kDa, produced by the sequential hydrolysis of amyloid precursor protein (APP) by β-secretase and γ-secretase. The most common isoforms of Aβ are Aβ1-40 or Aβ40 and Aβ1-42 or Aβ42 with 40 and 42 amino acid residues, respectively. The enhanced hydrophobicity of Aβ42, compared with Aβ40, renders it intrinsically more susceptible to misfolding and subsequent aggregation. It can sequentially form soluble oligomers, insoluble fibrils, and eventually deposits as senile plaques. Studies have shown that soluble Aβ oligomer (AβO) is the key toxic agent that can cause the impairment of synaptic plasticity, the suppression of long-term potentiation, and the memory deficits [20]. Tau is a soluble microtubule-associated protein with a molecular mass ranging from 55 to 62 kDa. It is mainly distributed in the axons of neurons, maintaining the stability of cytoskeletons and promoting the assembly of microtubules. Under physiological conditions, Tau can dynamically regulate its binding to microtubules through the modification of phosphorylation, glycosylation, and oxidation. In AD, abnormal hyperphosphorylation will induce Tau to detach from microtubules, aggregate into soluble oligomers, and further form neurofibrillary tangles, leading to severe neurotoxicity [21]. In addition, the deposition of Aβ can directly trigger Tau hyperphosphorylation and exacerbate glutamatergic signaling disorders and calcium homeostasis imbalance via Fyn kinase, further accelerating pathological progression. Among numerous phosphorylation sites, p-Tau181, p-Tau217, and p-Tau231 exhibit the strongest correlation with AD pathology [22,23]. Notably, the levels of these phosphorylated Tau species are markedly upregulated in peripheral biofluids at early—and even presymptomatic—stages, demonstrating favorable diagnostic accuracy for AD. In addition, other isoforms including p-Tau205 and p-Tau212 also show strong potential for distinguishing AD from other neurodegenerative diseases and show potential in disease staging or joint diagnosis [23].

The concentrations of Aβ and Tau proteins are highest in CSF that can directly contact the brain’s extracellular space [24]. For example, the level of Aβ42 in the CSF of AD patients is usually below 500 pg/mL, while the level in healthy individuals is approximately 800 pg/mL. The level of total Tau in AD patients reaches about 850 pg/mL, which is approximately twice that of healthy controls. The level of p-Tau181 in AD patients is approximately 95 pg/mL, while in the healthy controls it is approximately 48 pg/mL. Although CSF samples offer high diagnostic accuracy for AD diagnosis, the invasiveness of lumbar puncture limits their clinical application [25,26,27]. In contrast, plasma samples can be obtained via routine blood collection. However, AD biomarkers must cross the blood-brain barrier to enter the bloodstream, resulting in reduced concentrations typically down to pg/mL or even fg/mL levels [24,28,29]. For example, the level of Aβ42 in the plasma of AD patients can drop below 20 pg/mL, and that of p-Tau181 varies in the range of approximately 10~25 pg/mL. Saliva and urine can provide non-invasive sample collection. However, the concentrations of biomarkers in saliva and urine are even lower, with large individual differences and poor stability. For instance, the level of total Tau in saliva usually ranges from 10 to 20 pg/mL, with substantial overlap between healthy individuals and patients, making it extremely difficult to establish a diagnostic cut-off value.

Aβ42/Aβ40 and different subtypes of p-Tau proteins such as p-Tau217, p-Tau181, and p-Tau231 have been proven to be effective blood indicators for early screening of AD. Among these preferred protein biomarkers, the level of p-Tau217 and the ratio of Aβ42/Aβ40 or p-Tau217/Aβ42 are particularly recommended as the most valuable indicators for AD staging or prognosis [8,30]. For mixed-type AD patients, comprehensive analysis should be conducted by integrating clinical evaluation, neuroimaging findings, and other non-AD-specific biomarkers to avoid missed diagnoses or misdiagnoses due to atypical changes in the concentrations of core AD biomarkers.

## 3. Optical Methods

Among emerging detection techniques, optical methods are highly valued for their ability to achieve efficient, specific, and minimally invasive target detection. They play a pivotal role in early diagnosis and real-time monitoring of disease progression, and exhibit high sensitivity, low cost, and low detection limit. Some of them show faster response than electrochemical techniques. For example, colorimetric and fluorescent assays can measure the changes in color/fluorescence intensity, enabling rapid and simple detection. Usually, there are mainly two detection types for optical methods: direct binding assays and sandwich assays. The signals of direct binding assays originate from the analyte-receptor interactions. In contrast, signaling tags are usually used in sandwich assays to generate corresponding signals that can be determined through colorimetric, fluorescence, or luminescent techniques.

### 3.1. Colorimetric Assays

Colorimetric assays are highly favored owing to their inherent advantages of cost-effectiveness and unnecessary requirement for sophisticated equipment. According to the type of sensing element, colorimetric assays can be categorized into several types, including aptasensors, immunoassays, and enzymatic methods with aptamers, antibodies, and enzymes as the receptors, respectively. Traditional colorimetric methods typically involve enzymatic chromogenic systems. For instance, horseradish peroxidase (HRP) is usually employed to catalyze the oxidation of 3,3′,5,5′-tetramethylbenzidine (TMB) by hydrogen peroxide, resulting in observable color changes [31]. Compared with natural enzymes, nanozymes exhibit higher stability, lower cost, and comparable catalytic activity. In addition, nanomaterials have been employed as signal indicators or carriers in colorimetric assays, greatly improving detection sensitivity. Nanomaterials can also be modified with aptamers, antibodies, or other recognition elements to exhibit unique physical, chemical, and optical properties. For colorimetric detection of AD biomarkers, the sensing strategies mainly include enzyme- or nanozyme-mediated chromogenic reactions and SPR changes induced by the aggregation or dispersion of nanoparticles. Through these mechanisms, molecular recognition events can be converted into observable color outputs, enabling straightforward and sensitive assays of AD biomarkers from blood samples.

**Figure 1:**
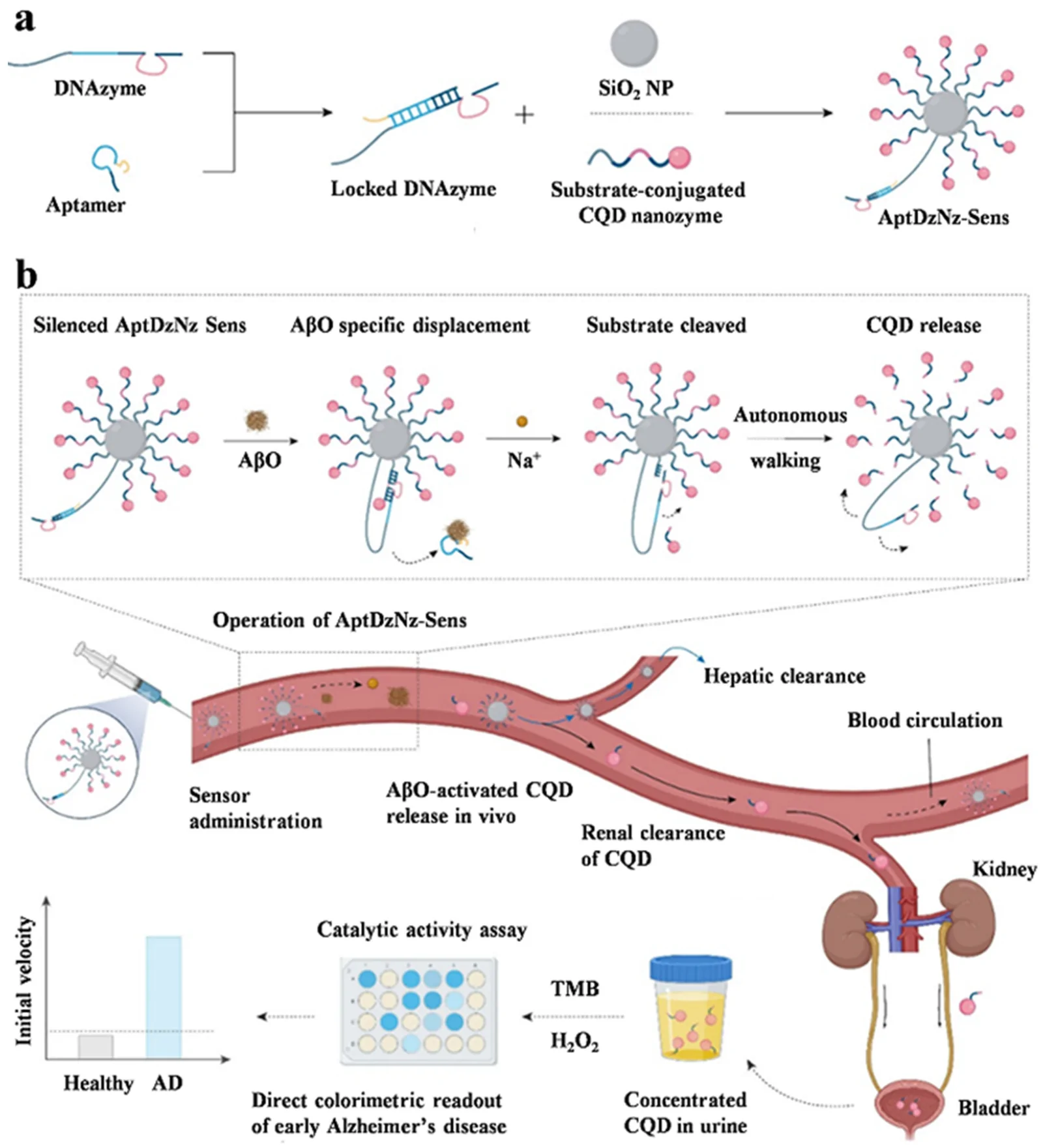
Schematic illustration of synthesis of AptDzNz-Sens (**a**) and colorimetric urinalysis of AD (**b**). The DNAzyme contains a spacer with 43 thymine residues (gray blue), a domain with 12 nucleotide residues (light blue), and a catalytic core (pink) flanked by two DNA domains (dark blue). The aptamer contains an AβO-binding domain (yellow), a domain with 12 nucleotide residues (light blue), and a sequestering domain of 7 nucleotide residues (dark blue). The substrate contains a spacer with 10 thymine residues (gray blue) and two deoxyribonucleotide domains that can hybridize with the DNAzyme (dark blue), which are separated by a strand of deoxyribonucleotides (pink) with an enzyme cleavage site (a ribonucleotide) in the middle. Reprinted with permission from ref. [36].

Through the chromogenic reactions, enzymes and nanozymes used for the determination of Aβ and Tau proteins mainly include HRP [32,33], urease [34], hemin/G-quadruplexes [35], DNAzyme [36], Au@Pt and Ni@Pt nanozymes [37,38], aptamer-templated DNA-Ag/Pt nanoclusters [39], and porous bimetallic ZnO-Co_3_O_4_ nanocages [40]. Analytical performances for the detection of different targets based on the chromogenic reactions are shown in Table 1. An excellent example is that Chen et al. developed a universal and modular artificial probe for the detection of AβO (Figure 1) [36]. This probe featured an innovative three-component design of aptamer, DNAzyme, and nanozyme. Silica nanoparticles (SiO_2_ NPs) served as the carriers for covalent immobilization of AβO-specific aptamers, DNAzymes, and carbon quantum dots (CQDs) with peroxidase (POD)-like activity. The specific aptamer-AβO recognition reaction induced conformational change in aptamers to activate DNAzymes, leading to the subsequent cleavage of substrates and the release of large numbers of CQDs. The released CQDs could catalyze the oxidation of colorless TMB by hydrogen peroxide to form blue products, allowing for the visual detection of AβO. This strategy has been used for early diagnosis of AD by urine testing, and its sensitivity is 67 times higher than that of conventional ELISA. Multiple assessments in vivo confirmed the favorable biocompatibility and reliable ability of this method to distinguish AD model mice from healthy individuals. This modular sensing strategy opens up a novel non-invasive direction for the early diagnosis of AD.

**Figure 2:**
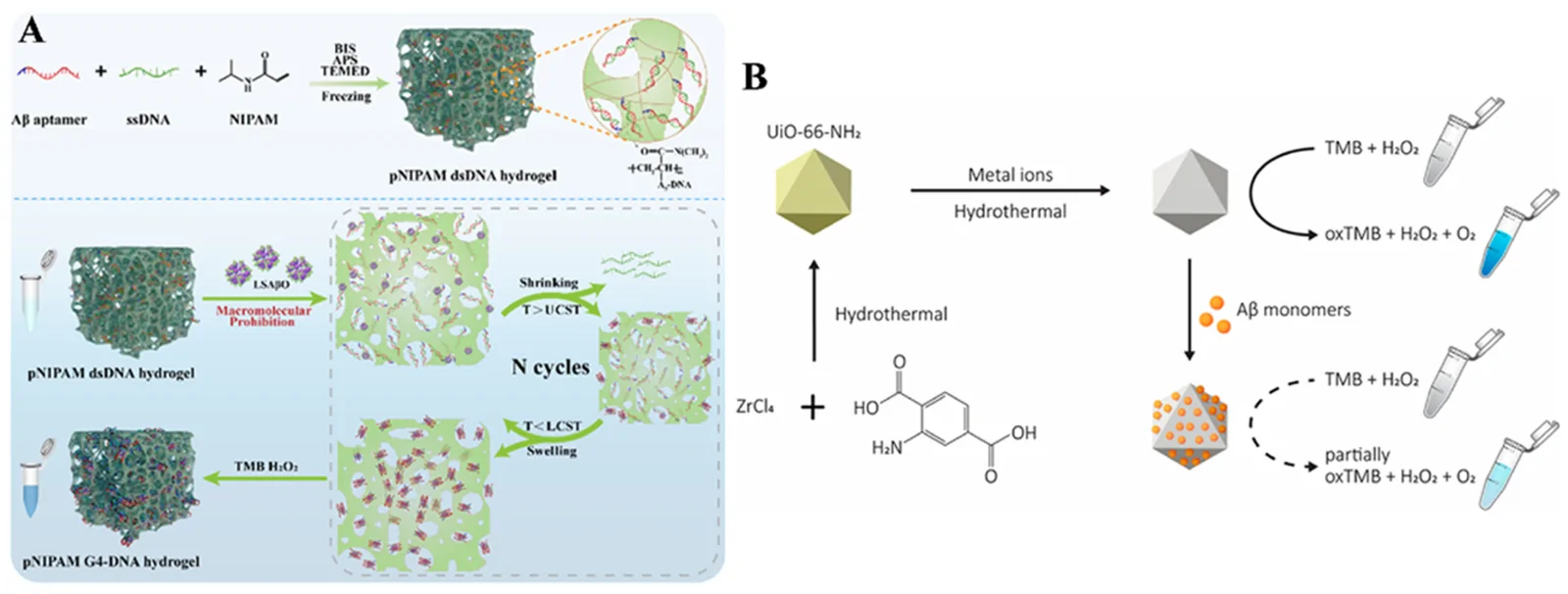
(**A**) Schematic illustration of the preparation of the functionalized pNIPAM-DNA hydrogel and the colorimetric signal amplification platform for LSAβO detection. Reprinted with permission from ref. [41]. (**B**) Schematic illustration of synthesis of metal-loaded UiO-66-NH_2_ and Aβ42 monomer detection by inhibiting TMB oxidation. Reprinted with permission from ref. [43].

Aptasensors with synthetic oligonucleotides or peptides as receptors to achieve highly specific target recognition have been widely developed for the detection of AD biomarkers. Chen et al. fabricated a temperature-responsive pNIPAM-DNA hydrogel platform for colorimetric detection of low-molecular-weight soluble *β*-amyloid oligomers (LSA*β*O) (Figure 2A) [41]. The hydrogel achieved target enrichment via reversible swelling and shrinking under temperature control, triggering the formation of a G-quadruplex DNAzyme upon specific binding with LSA*β*O. The formed DNAzyme catalyzed TMB oxidation to produce visible color signals. Benefiting from the pore size screening effect of the hydrogel, the biosensor effectively excluded large amyloid aggregates and other interferents. It showed a linear detection range of 0.1–7.5 nM with a detection limit of 50 pM. Validated with artificial and clinical CSF samples, the method showed favorable accuracy and reproducibility. Featuring simple operation, low cost, and short detection time, the platform is suitable for high-throughput preliminary screening and early auxiliary diagnosis of AD. However, the inherent separation of recognition elements and signal transduction components in traditional strategies may increase the detection complexity and economic cost [42]. A substantially integrated platform capable of simultaneous recognition, signal transduction, and intrinsic amplification will represent a decisive breakthrough in point-of-care diagnostic technologies. For this consideration, Fan et al. synthesized a variety of metal-functionalized UiO-66-NH_2_ metal–organic framework (MOF) nanozymes and found that the platinum-loaded MOF showed optimal peroxidase-like catalytic performance (Figure 2B) [43]. A colorimetric sensing strategy was then established on the basis of the competitive binding between Aβ42 and MOF. The sensor featured favorable anti-interference capability and could inhibit the aggregation of Aβ peptides. The detection limit was found to be 2.76 nM for the assay of Aβ42 in pure solution and 4.65 nM in diluted CSF. Due to its low cost and ease of operation, this colorimetric platform is a promising candidate for laboratory analysis and bedside AD screening.

**Figure 3:**
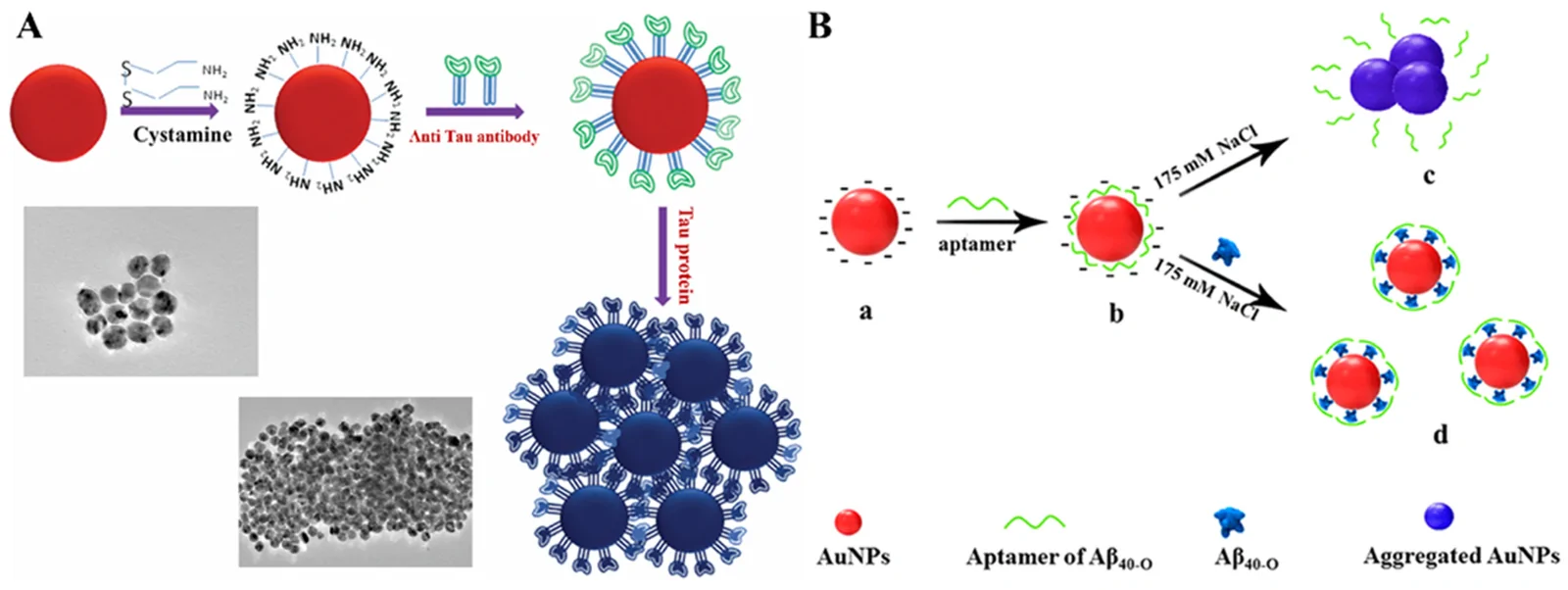
(**A**) Schematic representation for synthesis of monoclonal anti-Tau antibody-conjugated AuNPs and sensing of Tau protein. Reprinted with permission from ref. [45]. (**B**) Schematic illustration of aptasensor for the detection of LS-Aβ40-O under high-salt conditions: (a) Negatively charged AuNPs, (b) AuNPs modified with Apt, (c) aggregation of Apt@AuNPs at a high salt concentration of 175 mM NaCl, and (d) disaggregation of Apt@AuNPs in the presence of LS-Aβ40-O at a high salt concentration of 175 mM NaCl. Reprinted with permission from ref. [47].

In addition to the abovementioned chromogenic reactions catalyzed by natural or artificial enzymes, another strategy for colorimetric analysis is based on the analyte-regulated nanoparticle aggregation. Gold and silver nanoparticles (AuNPs and AgNPs) exhibit unique optical properties due to their SPR effect. When these nanoparticles transition from a dispersed state to an aggregated state in solution, a distinct color change occurs. Specific target molecules (e.g., Aβ peptides or Tau protein) can modulate nanoparticle assembly, enabling enzyme-free colorimetric detection, including antibody-modified AuNPs or AgNPs [44,45,46], aptamer-functionalized AuNPs [47,48,49], Cu^2+^-mediated AuNPs [50], and AuNPs-functionalized microneedles [51]. Typically, Neely et al. for the first time reported the colorimetric detection of Tau protein using monoclonal anti-Tau antibody-coated AuNPs (Figure 3A) [45]. The detection limit of 1 pg/mL is about two orders of magnitude lower than the cut-off value (195 pg/mL) for Tau protein in CSF. In addition, Deng et al. developed a AuNP-based colorimetric aptasensor for the detection of low-mass, soluble Aβ40 oligomers (LS-Aβ_40-O_) (Figure 3B) [47]. The target-specific aptamer serving as the recognition element for LS-Aβ_40-O_ could adsorb on the surface of AuNPs. A high-salt condition was used to induce the aggregation of aptamer-modified AuNPs. The formation of LS-Aβ_40-O_-aptamer complexes on the nanoparticle surface enhanced the salt tolerance of AuNPs. This method showed a linear range of 35~700 nM in the presence of 175 mM NaCl with a detection limit of 10 nM for LS-Aβ_40-O_. It was further used to distinguish CSF samples from healthy persons and AD patients. However, the aggregation-based colorimetric methods are rarely applied to the detection of blood-based AD biomarkers, likely due to their low sensitivity and potential interference from unknown blood components.

**Figure 4:**
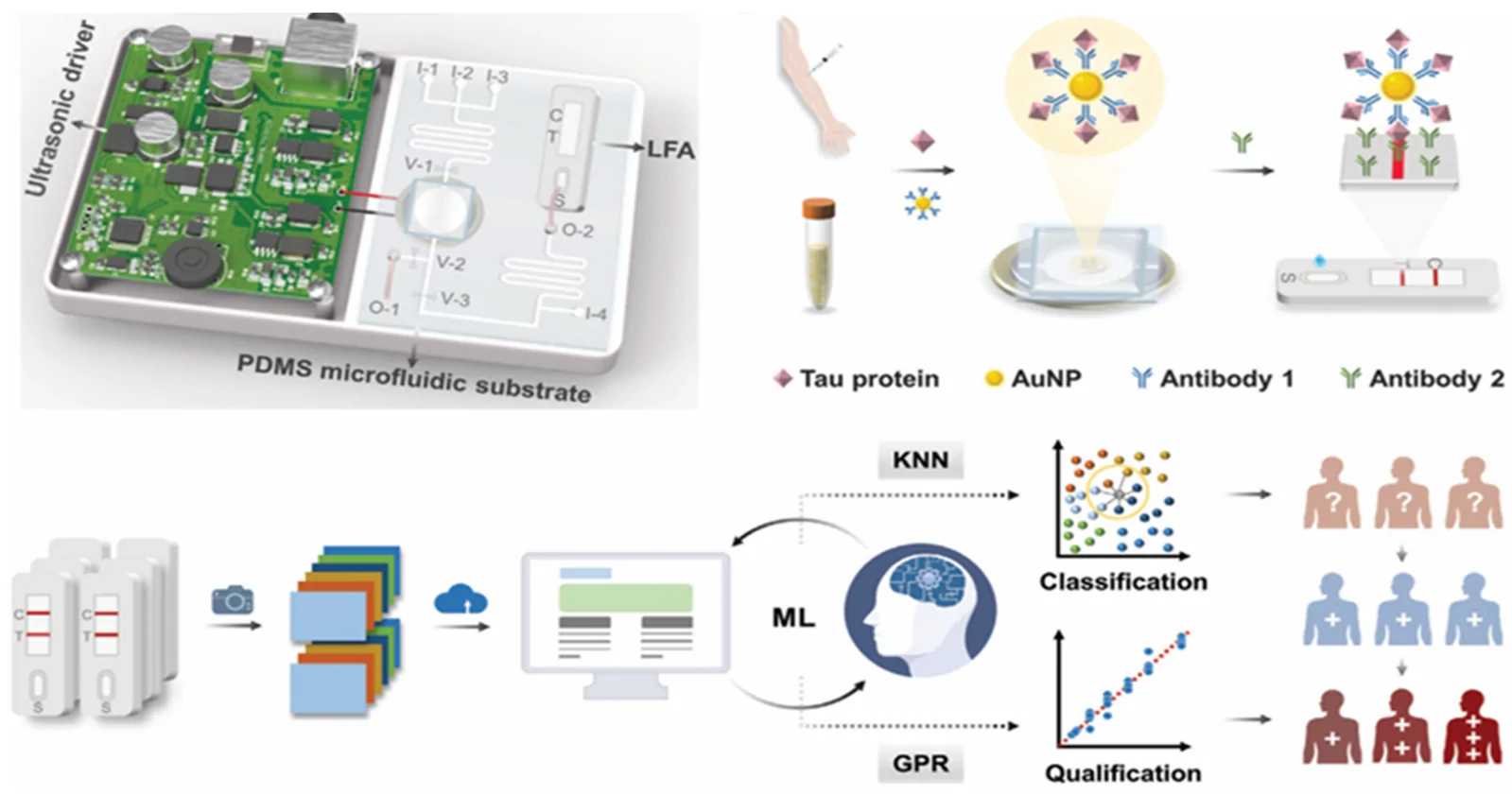
Schematic illustration of the developed machine learning-optimized lateral flow assay with ultrasound enrichment for Tau protein biosensing in AD. Reprinted with permission from ref. [52].

Researchers have been exploring integrated detection methods and signal enhancement strategies to overcome the complexity of blood samples and achieve visualization of ultra-low abundance biomarkers. Lateral flow and paper-based optical immunoassays can detect most of the core AD biomarkers and hold promise for point-of-care applications [52,53,54]. A lateral flow assay based on AuNPs is one of the most promising tools for rapid diagnosis of diseases. Wang et al. developed an ultrasound-enhanced, machine learning-optimized colorimetric flow assay platform for sensitive detection of Tau (Figure 4) [52]. The method achieved a detection limit of 10.3 pg/mL, a linear range of 0.02–4 ng/mL, and a total assay time of approximately 20 min. The device was integrated with a portable ultrasonic actuator to rapidly enrich particles through ultrasound, which is crucial for improving sensitivity through sample pre-enrichment, followed by machine learning-assisted classification and prediction of amplified colorimetric signals. Further point-of-care testing on human plasma confirmed the clinical potential of the flow assay method. It could effectively classify and detect Tau in undiluted serum, achieving an average classification accuracy of 98.11% and an average prediction accuracy of 99.99%.

**Table 1:** Performances of colorimetric platforms for the detection of Aβ and Tau.

Reaction	Signal Reporter	Target	Detection Limit	Linear Range	Sample	Ref.
Catalysis	Antibody/AuNPs-HRP	p-Tau^396,404^	24 pg/mL	1.5–10^6^ pg/mL	Blood (6 HI + 10 AD)	[32]
	Aptamer/Ni-HRP	Aβ40	0.22 nM	0–10 nM	Spiked human serum	[33]
	Aptamer/urease	AβO	2.8 fM	5 fM–10 nM	Spiked artificial CSF	[34]
	Aptamer/DNAzyme	AβO	0.23 pM	0.3472–694.44 pM	Spiked mice CSF	[35]
	Aptamer/DNAzyme	AβO	2.8 pg/mL	60–1200 pg/mL	Mice urine (14 HI + 11 AD)	[36]
	Antibody/Ni@Pt	Aβ42	42 particles/μL	10–10^5^ particles/μL	Mice plasma (6 HI + 6 AD)	[37]
	Aptamer/Au@Pt	Aβ42	72 particles/μL	100–10^4^ particles/μL	Mice plasma (7 HI + 3 AD)	[38]
	Aptamer/Ag/Pt NCs	AβO	1.22 nM	0–0.7 μM	Spiked human serum	[39]
	ZnO-Co_3_O_4_ nanocages	Aβ40	3.5 nM	5–150 nM	Spiked mice CSF	[40]
	pNIPAM-aptamer	LSAβO	50 pM	0.1–7.5 nM	Spiked human CSF	[41]
	Pt-loaded MOF	Aβ42	4.65 nM	0.75–25 nM	Spiked artificial CSF	[43]
Aggregation	Antibody–AgNPs	Aβ40/42	86 pM	0.25–150 nM	Spiked human serum	[44]
	Antibody–AuNPs	Tau	1 pg/mL	5–350 ng/mL	Spiked human CSF	[45]
	Antibody–AuNPs	Aβ42	2.3 nM	7.5–350 nM	Spiked human serum	[46]
	Aptamer–AuNPs	LSAβO	10 nM	35–700 nM	Spiked human CSF	[47]
	Aptamer–AuNPs	AβO	3.03 nM	10–100 nM	Spiked artificial CSF	[48]
	AuNPs/AgNPs/Cu^2+^	Aβ40/42	50 nM	50–500 nM	Spiked human plasma	[50]

Abbreviation: AuNPs, gold nanoparticles; HRP, horseradish peroxidase; NCs, nanocubes; pNIPAM, poly(N-isopropylacry lamide) hydrogel; AgNPs, silver nanoparticles; p-Tau^396,404^, phosphorylation of Tau at Ser 396, 404; LSAβO, low-mass soluble β-amyloid oligomer; CSF, cerebrospinal fluid; EVs, neuronal-derived extracellular vesicles; HI, healthy individual.

### 3.2. Fluorescence Assays

**Figure 5:**
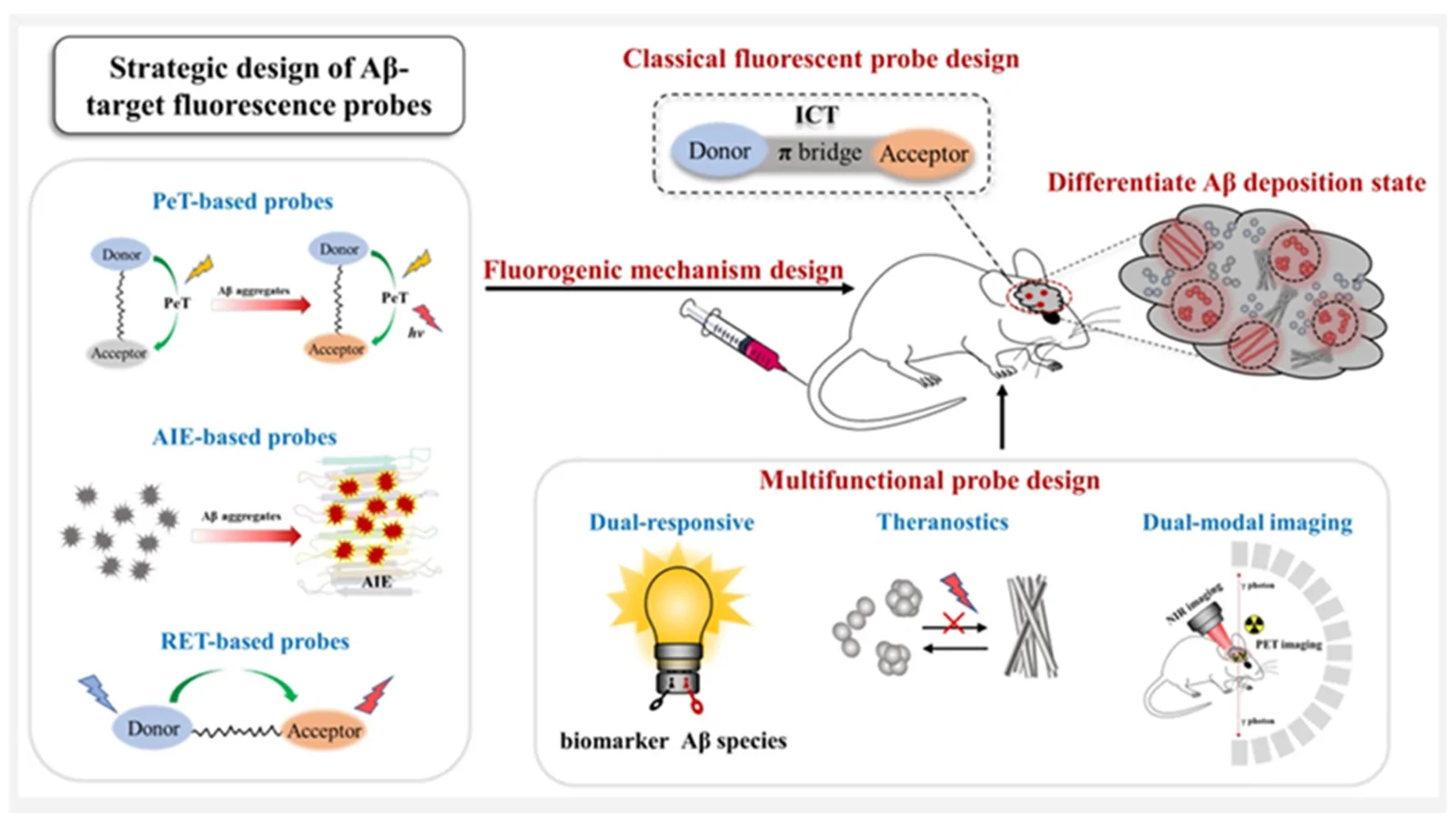
Strategies for designing fluorescence probes to image Aβ plaques. Reprinted with permission from ref. [56].

Fluorescence-based protein detection platforms offer numerous advantages, including ultra-high sensitivity, rapid signal response, and relatively simple operation. Fluorescence imaging has attracted the attention of researchers to develop various probes for early diagnosis of AD. Many reviews have focused on different design strategies for imaging of Aβ and Tau aggregates with small molecule fluorescence probes through the regulation mechanisms of photoinduced electron transfer (PeT), aggregation-induced emission (AIE), resonance energy transfer (RET), internal charge transfer (ICT) and so on (Figure 5) [55,56,57,58], paving the way for the future development of novel diagnosis tools. However, the picomolar or sub-picomolar abundance of Aβ, Tau, and their associated species in blood is much lower than the detection limit of conventional organic fluorescence probes, rendering reliable quantification exceedingly difficult. Two principal approaches have been pursued to address this bottleneck and enhance overall performance. The first is to upgrade fluorescence detection equipment and use large or advanced optical systems to capture weak fluorescence signals. The second is to develop advanced fluorescence methods and amplification technologies to enhance the signal intensity and stability, enabling ultra-sensitive detection of AD-related blood biomarkers. Given the expensive and complex operation as well as the bulky nature of sophisticated fluorescence equipment, this section only provides a focused summary of the latest research on detecting protein biomarkers based on nanoprobe engineering and signal amplification technology.

**Figure 6:**
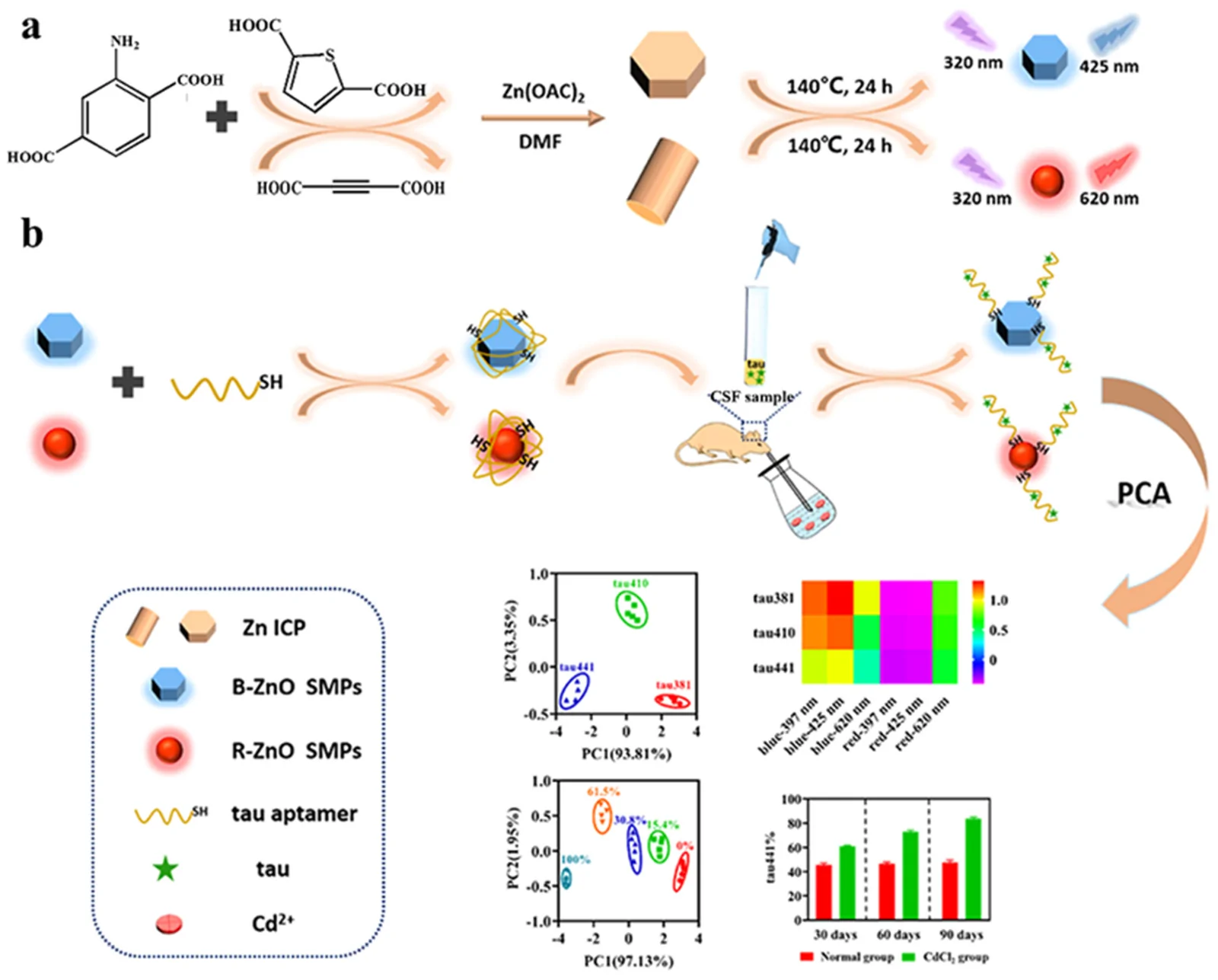
(**a**) Schematic illustration of synthesis of B- and R-ZnO SMPs based on the post-treatment strategy of Zn-ICP precursors. (**b**) Schematic illustration of the sensor array for identifying different Tau isoforms and in vivo monitoring CSF Tau441% upon Cd^2+^ exposure. Reprinted with permission from ref. [70].

The use of fluorescently labeled recognition elements to recognize targets and generate signals is a traditional fluorescence detection technique. In this approach, fluorescence tags are usually conjugated to biorecognition elements, allowing for qualitative and quantitative detection of Aβ, Tau, and a range of other AD-related biomarkers. This method is simple to operate and allows for direct optical readout, making it highly suitable for routine laboratory analysis. FRET effect arises from non-radiative energy transfer. When the donor–acceptor distance is shorter than ~10 nm, the fluorescence of the donor will be quenched by the acceptor. Once the distance is beyond this range, the FRET process will be blocked, and the donor fluorescence will be restored. With the continuous advancements of probe design, nanotechnology, and signal transduction mechanisms, various fluorescence detection platforms have emerged. Recently, FRET-based fluorescence sensing platforms have also been widely developed for the detection of AD protein biomarkers with nanomaterials as the quenchers (Table 2) [59,60,61,62,63,64,65,66,67,68,69,70]. Such methods typically involve two steps: adsorption of a fluorescently labeled probe onto the nanoquencher surface results in fluorescence quenching, and binding of the target with the probe restores the fluorescence. Based on this principle, our groups have achieved the detection of AβO with fluorescently labeled aptamers or peptides as the detection probes and porphyrin-substituted phenylalanine–phenylalanine nanoparticles, polydopamine nanospheres, and graphene oxide as the nanoquenchers [71,72,73]. More interestingly, the nanoquenchers could be used as nanodrugs to inhibit the aggregation of Aβ monomers and dissolve the aggregates. In addition, Liu et al. developed a sensor array to monitor Tau protein in CSF and clarified the correlation between the exposure of cadmium ions and the pathological aggregation of Tau in AD (Figure 6) [70]. Two types of zinc oxide (ZnO) submicron particles (SMPs) were synthesized with distinct luminescent properties, namely blue-emitting B-ZnO and red-emitting R-ZnO. The authors investigated the varied spectral responses of aptamer-modified B-ZnO and R-ZnO toward different Tau isoforms under ultraviolet and visible light irradiation. With the assistance of principal component analysis (PCA), the unique spectral fingerprint of the sensor array enabled simultaneous quantification of Tau381, Tau410, and Tau441 for the first time. The biosensor was further applied to track the dynamic change in Tau 441/Tau381 ratio (Tau441%) in the CSF of AD rats after exposure to cadmium ions.

**Figure 7:**
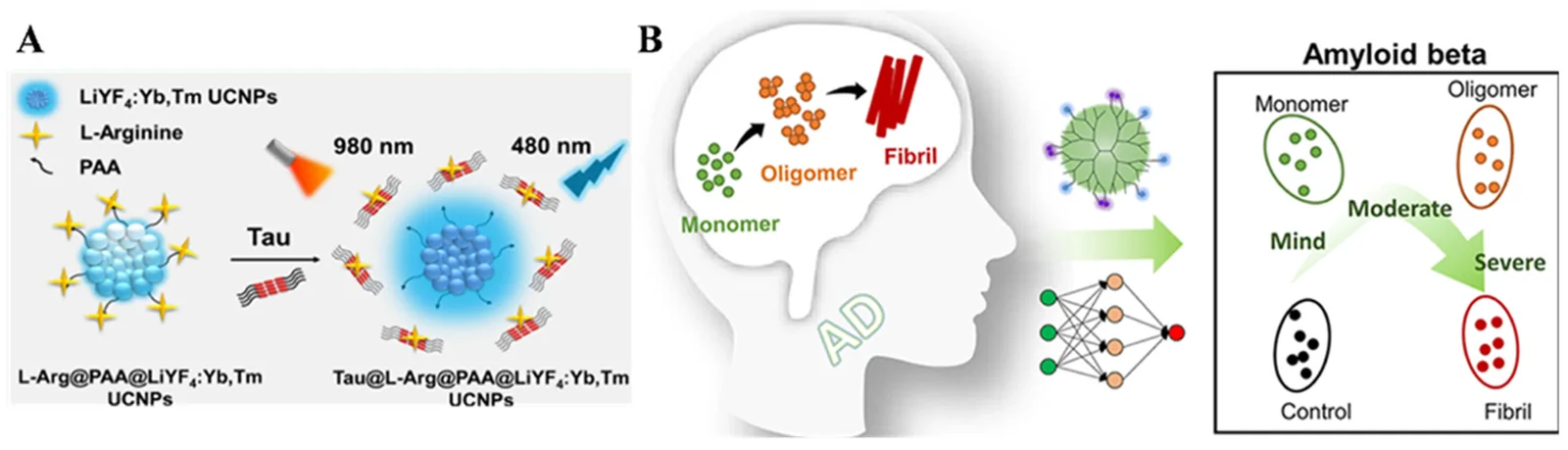
(**A**) Schematic illustration of the design of the L-arginine-ligated PAA@LiYF_4_:Yb,Tm photon upconversion nanoprobe for the detection of Tau peptide. Reprinted with permission from ref. [75]. (**B**) Schematic illustration of the PAMAM dendrimer-based sensor array for the identification of multiple Aβ40/Aβ42 aggregates. Reprinted with permission from ref. [76].

After being incorporated with small functional groups or metal species, sensing materials can be designed to exhibit specific recognition and signal transduction capabilities. These attributes have enabled the simple and rapid detection of AD biomarkers with high sensitivity and selectivity [74,75,76]. For example, Abraham et al. designed an antibody-free nanoprobe (L-Arg@PAA@LiYF_4_:Yb,Tm) for the detection of Tau protein. Under the excitation of a 980 nm laser, the synthesized upconversion nanoparticles emitted blue fluorescence at 480 nm (Figure 7A) [75]. The nanoparticles were further surface-functionalized with negatively charged polyacrylic acid, and their upconversion luminescence was quenched by positively charged L-arginine via electrostatic interactions. Binding of Tau protein to the guanidine group of L-arginine restored the luminescence of nanoprobes. The method achieved a detection limit of 5.01 pg/mL and a linear range of 49.18–477.7 pg/mL. It exhibited good selectivity with minimal interference from competing proteins and other biomarkers, with a recovery rate of 96–105% in spiked human serum samples. In addition, Xu et al. developed a fluorescence sensor array composed of three modified polyamide dendrimers (Figure 7B) [76]. This array has distinguished various proteins with 100% accuracy, demonstrating the rationality of this design. Using machine learning algorithms, the robust ternary array enabled parallel detection of multiple Aβ40/Aβ42 aggregates in the presence of different interfering substances, serum, and CSF, with a detectable concentration of 0.5 μM, showing great potential for AD diagnosis.

**Figure 8:**
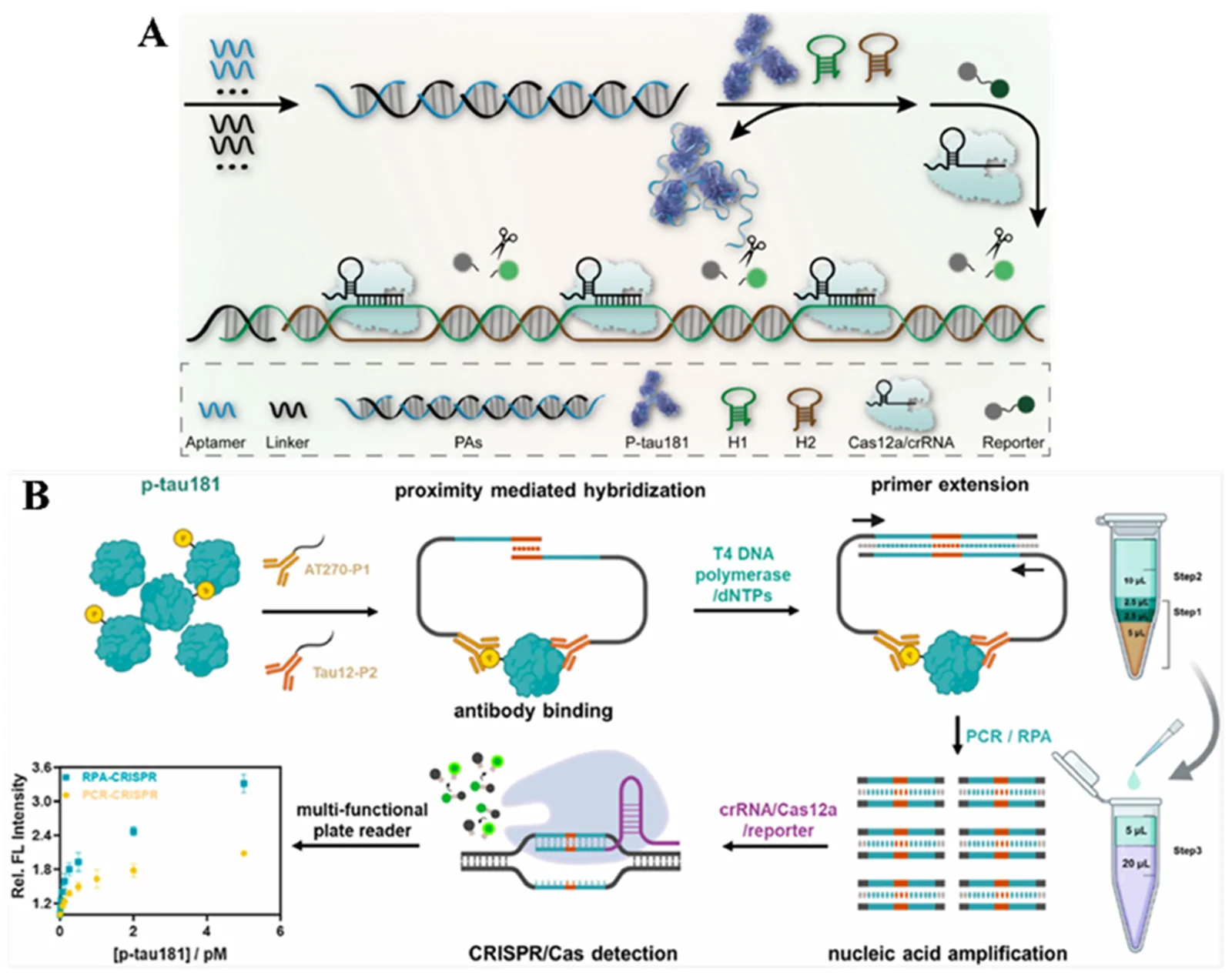
(**A**) Schematic illustration of a fluorescence aptasensor for the detection of p-Tau 181 protein by coupling PAs structure, HCR and CRISPR-Cas12a system. Reprinted with permission from ref. [77]. (**B**) Schematic illustration of the principle and workflow of the PEA-PCR/RPA-CRISPR/Cas assay for the homogeneous detection of p-Tau181. Reprinted with permission from ref. [78].

Fluorescence probes are susceptible to background interference and photobleaching, and conventional fluorescence methods often lack sufficient sensitivity to quantify blood-based biomarkers with ultralow concentrations. To address these limitations, advanced fluorescence nanomaterials have been developed and further integrated with ratiometric or fluorescence “turn-on–off–on” detection modes [77,78,79]. These strategies substantially enhance the overall analytical performance (e.g., sensitivity, stability, and reproducibility), making them well suited for reliable quantification of blood-based AD biomarkers. For instance, based on the fact that FRET-based ratiometric or fluorescence-switching sensing systems can provide self-calibration and improve analytical accuracy, Li et al. designed an ultrasensitive fluorescence aptasensor for p-Tau181 detection by integrating multivalent aptamers, hybridization chain reaction (HCR), and CRISPR-Cas system (Figure 8A) [77]. Due to the synergistic effects of signal amplification, high specificity of aptamer, and excellent cleavage activity of Cas, the biosensor achieved a wide linear range (0.1–10^6^ pg/mL) and a low detection limit (0.069 pg/mL) and exhibited high selectivity and strong anti-interference capability. It can effectively determine p-Tau181 in serum and artificial CSF, showing great promise for early diagnosis of AD. In addition, Liu et al. developed a sensitive method for fluorescence assay of p-Tau181 by combining proximity extension assay (PEA) with PCR/RPA and CRISPR/Cas12a systems (Figure 8B) [78]. The binding of two PEA probes to a single p-Tau181 molecule triggered probe hybridization and extension, converting the protein assay into amplifiable nucleic acid detection. The resulting DNA was quantified by the signal amplification of PCR/RPA and Cas12a trans-cleavage. The PEA-PCR-CRISPR/Cas and PEA-RPA-CRISPR/Cas systems achieved detection limits of 149 fM (6.8 pg/mL) and 45.4 fM (2.1 pg/mL), respectively. The accuracy of the PEA-RPA-CRISPR/Cas assay in human serum was validated against commercial ELISA kits.

Fluorescence immunoassays are a type of sensing method performed on biological samples to detect specific antigens in any biological specimen or sample and vice versa. The recognition antibodies can be labeled with fluorescence dyes, enzymes, and quantum dots (QDs) for signal readout with a direct or sandwich immunoreaction as an alternative staining procedure [80,81,82,83,84,85,86,87]. For example, Gagni et al. developed a highly sensitive fluorescence immunoassay platform to detect Aβ42 at 73 pg/mL in artificial CSF by coating silicon chips with a terpolymer of dimethylacrylamide, 3-(trimethoxysilyl)propyl methacrylate, and N-acryloyloxy succinimide [88]. Alishahi et al. successfully detected five AD blood biomarkers (Aβ40, Aβ42, p-Tau, NfL, GFAP) using this technique, demonstrating multiplexing advantages [89]. However, these methods have some obvious limitations, such as insufficient sensitivity for accurate quantification of low-abundance biomarkers, low automation, and long reaction times, restricting their clinical screening application. In addition, they involve multiple detection steps including washing, separation, incubation, and fluorescence detection or imaging.

**Figure 9:**
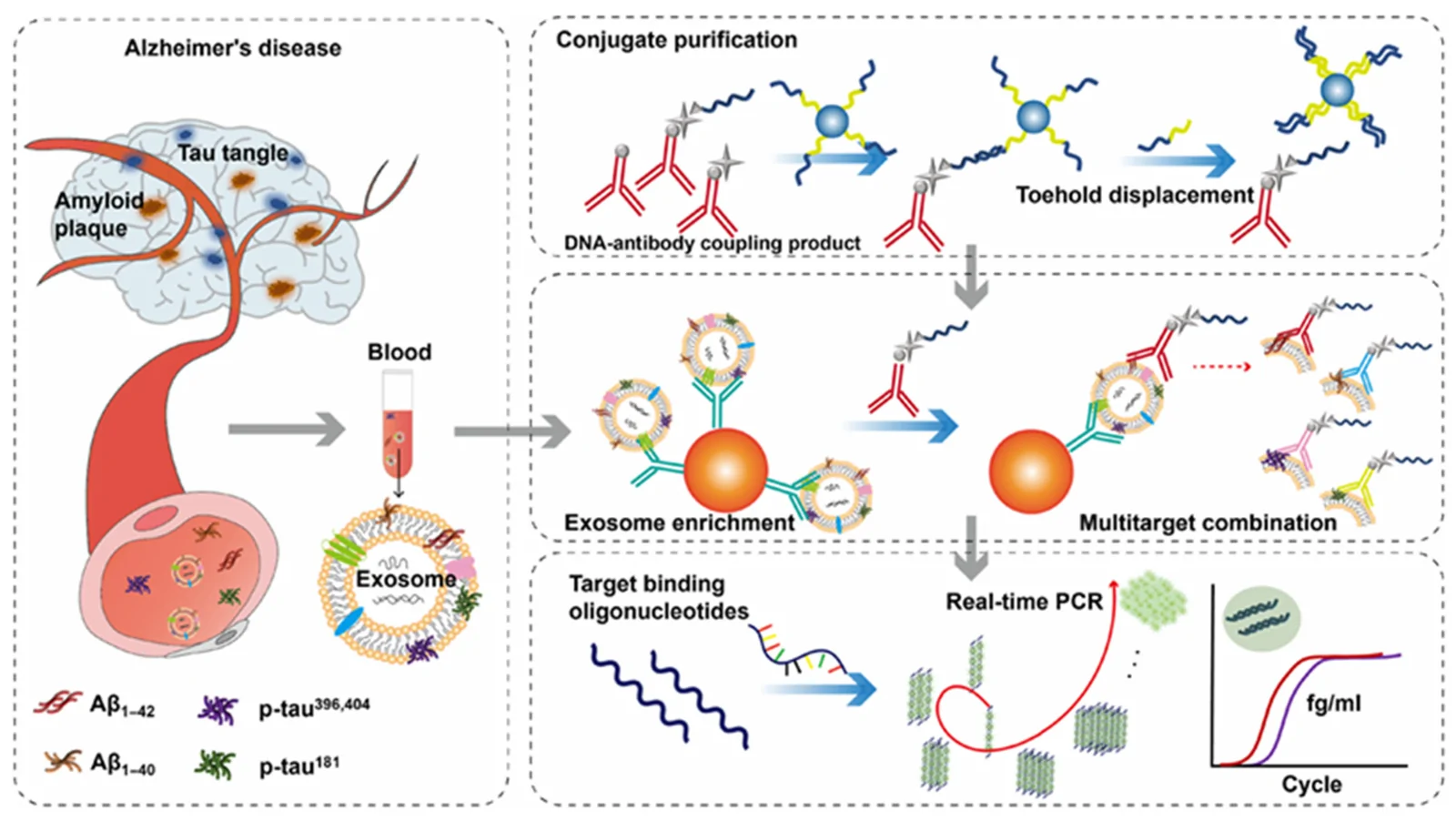
Schematic illustration of the iMEP assays by integrating the immunomagnetic enrichment of blood exosomes, immunorecognition of exosomal biomarkers, and real-time PCR. Reprinted with permission from ref. [94].

**Figure 10:**
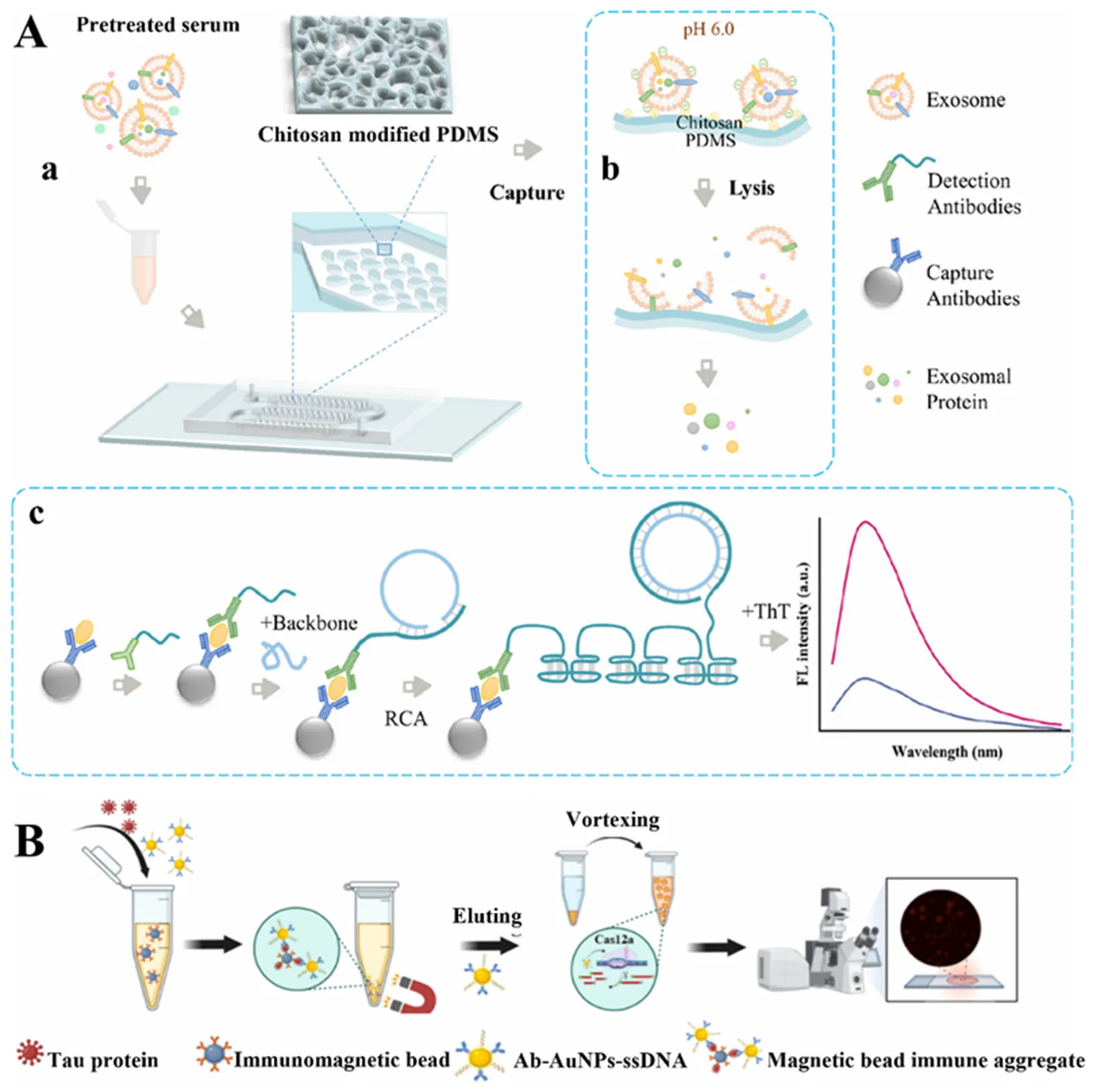
(**A**) Schematic illustration of the 3D drop-shaped porous microfluidic chip (**a**), in situ lysis of exosome on the microfluidic chip (**b**), and double antibody sandwich recognition and RCA signal amplification (**c**). Reprinted with permission from ref. [95]. (**B**) Schematic illustration of CRISPR-AD for the detection of protein biomarkers. Reprinted with permission from ref. [97].

Recently, researchers have developed various techniques to improve detection sensitivity by combining fluorescence immunoassays with magnetic separation and flow cytometry. Target proteins can be captured by antibody-coated plates, microspheres, or magnetic beads [90,91,92]. These methods offer high-throughput, multi-parameter simultaneous detection, providing important technical support for developing multiple assays of AD biomarkers. DNA amplification refers to the replication of a specific DNA sequence to increase its copy number. DNA-amplified techniques have been integrated with fluorescence immunoassays to significantly improve detection sensitivity and efficiency, including rolling circle amplification (RCA), polymerase chain reaction (PCR), primer exchange reaction, and CRISPR/Cas system [93,94,95,96]. Typically, Hu et al. developed an immunomagnetic exosome polymerase chain reaction (iMEP) platform for the rapid detection of Aβ (Aβ40, Aβ42) and phosphorylated Tau (p-Tau^396,404^, p-Tau181) in human blood exosomes (Figure 9) [94]. In this study, toehold-mediated DNA strand displacement was used to purify and enrich DNA-antibody conjugates. Combining the high specificity of sandwich ELISA with the signal amplification capability of PCR, the iMEP platform could readily determine the target at a concentration down to 10 fg/mL. This method enabled the selective identification of multiple AD-associated biomarkers in blood exosomes and differentiated clinically diagnosed AD patients from healthy individuals by measuring the target levels with 95% sensitivity and specificity. In addition, Xu et al. reported a sensitive system for the quantitative analysis of Aβ42 and p-Tau181 in serum exosomes by combining droplet-shaped porous microfluidic chips with RCA immunomagnetic beads (Figure 10A) [95]. This method realized non-invasive early diagnosis of AD with a femtomolar detection limit, outperforming traditional ultracentrifugation methods. The favorable sensitivity and specificity enabled it to distinguish patients in preclinical stages. Guo et al. developed a digital analytical platform named CRISPR-AD based on the CRISPR/Cas system for simultaneous detection of AD-associated proteins and microRNAs in peripheral blood (Figure 10B) [97]. Based on the collateral cleavage activity of Cas12a and Cas13a for cascaded signal amplification, this platform achieved extremely low detection limits (60 fg/mL for p-Tau217 and 0.5 fM for miRNA-34a). Clinical validation using plasma samples from AD patients, subjects with mild cognitive impairment, and healthy controls confirmed that the combined detection of dual biomarkers significantly improved diagnostic accuracy. The authors further integrated smartphone imaging and a deep learning algorithm to construct a portable detection module. This technology has enormous potential for on-site detection and early screening of AD.

**Table 2:** Performances of fluorescence assays of Aβ and Tau.

Method	System	Target	Detection Limit	Linear Range	Sample	Ref.
FRET	MB/Apt	Aβ42, Aβ40	110 fM, 300 fM	0.5–5 × 10^3^ pM, 1–10^4^ pM	Plasma (6 HI + 10 AD)	[59]
	Fe_3_O_4_/BaYF5:Yb UCNPs/Apt	AβO	36 pM	0.2–15 nM	Spiked artificial CSF	[60]
	FAM-Apt@PBNPs	AβO	1 nM	1–100 nM.	Human CSF (3 HI + 3AD)	[61]
	3W-CHA/PBPA/Apt	AβO	2.4 pM	5 pM–50 nM	Spiked artificial CSF	[62]
	MBs/DNA/ATMND	AβO	3.57 nM	0–70 nM	Phosphate buffer	[63]
	AuNPs@Apt@ RB/9-AC	AβO	147 nM	0.5–35 μM	Spiked human serum	[64]
	WS_2_NS@mAb@AuNCs	Tau	6.54 pg/mL	63.3–615.38 pg/mL	Spiked human serum	[65]
	FAM-Apt/AβO/cDNA-MBs	AβO	0.87 ng/mL	1.7–85.1 ng/mL	Spiked human plasma	[66]
	L-MOF/Apt-Au	AβO	0.3 pM	1–10,000 pM	Spiked human serum	[67]
	L-MOF/DNA/Apt-MBs	AβO	0.4 pg/mL	1–100,000 pg/mL	Spiked human serum	[68]
	ssDNA-Fe_3_O_4_@MB@MOF	AβO	3.4 fM	10 fM–10 μM	Spiked human serum	[69]
	B- and R-ZnO SMPs	Tau381, Tau410, tau441	Not reported	10–500 pg/mL	Spiked mice CSF	[70]
	Zn-FBIF	Aβ	3.22 nM	10–60 nM	Spiked bovine serum	[74]
	L-Arg@PAA@LiYF4:Yb,Tm UCNPs	Tau	5.01 pg/mL	49.18–477.7 pg/mL	Spiked human serum	[75]
	PAMAM	Aβ40/Aβ42	0.5 μM	Not reported	Spiked human serum	[76]
	PAs/HCR/CRISPR-Cas12a	p-Tau181	0.069 pg/mL	0.1–10^6^ pg/mL	Spiked human serum	[77]
	PEA-PCR-CRISPR/Cas, PEA-RPA-CRISPR/Cas	p-Tau181	45.4 fM, 87.1 fM	45.4–1500 fM	Spiked human serum	[78]
IMA	Cit@NaYF4,Yb,Tm UCNPs	Tau	3.4 pg/mL	64–377 pg/mL	Spiked human serum	[81]
	TYR/Anti-Tau@CuInS_2_/ZnS	Tau	9.3 pM	0.01–200 nM	Phosphate buffer	[82]
	Fe_3_O_4_@MoS_2_@Au-Ag/QD	Aβ42	11 ag/mL	0.1–10^4^ fg/mL	Spiked human serum	[84]
	Ag@SiO_2_@N,S-GQD	Aβ42	98 ng/mL	0.32–5000 pg/mL	Spiked human serum	[85]
	Au@CeO_2_/NGQDs/PNP	Tau-441	0.2 pg/mL	0.0005–10 ng/mL	Spiked human serum	[86]
	TriTDNs/MBs-Au/QDNBs	AβO	12.2 pM	0.02–100 nM	Spiked human serum	[87]
	DNA/SPOH/MNPs	Aβ42, Tau441, p-Tau181	8.4 fM, 4.3 fM, 3.6 fM	0–1000 fM	Spiked human serum	[91]
	Fe_3_O_4_@SiO_2_ NPs	AβO	25 fM	50–750 fM	Spiked human serum	[93]
	iMEP	Aβ40, Aβ42, p-Tau^396,404^, p-Tau181	0.01 pg/mL	10–10^5^ pg/mL	Spiked human serum	[94]
	MBs/RCA	Aβ42, p-Tau181	0.72 pM, 0.038 pM	10–10^6^ pg/mL, 10–10^7^ pg/mL	Serum (10 HI + 10 AD)	[95]
	AuNPs-CRISPR	p-Tau217, Aβ42	1 fM, 0.1 fM	Not reported	Plasma (47 HI + 66 AD)	[97]

Abbreviation: IMA, immunoassay; MB, molecular beacon; Apt, aptamer; UCNPs, upconversion nanoparticles; FAM-Apt, carboxyl fluorescein-modified aptamer; PBNPs, Prussian blue nanoparticles; 3W-CHA, three-way catalytic hairpin assembly; PBPA, palindromic sequence-based polymerase amplification; MBs, magnetic beads; ATMND, 2-amino-5,6,7-trimethyl-1,8-naphyridine; AuNPs, gold nanoparticles; RB, rose bengal; 9-AC, 9-anthracenecarboxylic acid; WS_2_ NS, tungsten disulfide nanosheets; AuNCs, gold nanoclusters; FAM-Apt, carboxyl fluorescein-modified aptamer; LMOF/Apt-Au, luminescent metal–organic framework carrying aptamer-modified AuNPs; B-ZnO SMPs, blue-color-emissive ZnO submicron particle; R-ZnO SMPs, red-color-emissive ZnO submicron particles; ZnFBIF, a bioinspired fluorescent probe based on self-assembled Zn-metal–organic framework with 2-methylimidazole and N-(6-(benzothiazol-2-yl)pyridin-3-yl)-5-(dimethylamino)naphthalene1-sulfonamide as the organic linker; L-Arg@PAA, arginine-ligated poly(acrylic acid); PAMAM, poly(amidoamine) dendrimers; CRISPR, clustered regularly interspaced short palindromic repeats; PAs, polyvalent aptamers structure; HCR, hybridization chain reaction; PEA, proximity extension assay; PCR, polymerase chain reaction; RPA, recombinase polymerase amplification; TYR, tyrosinase; QD-Ab, antibody-modified quantum dot; N, S-GQDs, N,S-doped graphene quantum dots; NGQDs, N-doped graphene quantum dots; PNP, p-nitrophenol; triTDNs, trivalent aptamer-modified tetrahedral DNA nanostructures TDNs; MBs-Au, magnetic beads-gold nanoparticles complexes; QDNBs, quantum dot nanobeads; SPOH, (E)-1-(2-hydroxyethyl)-4-(2-(9-(2-(2-methoxyethoxy)ethyl)-9H-carbazol-3-yl) vinyl)pyridin-1-ium chloride; MNPs, magnetic nanoparticles; iMEP, immunomagnetic exosome polymerase chain reaction; RCA, rolling circle amplification.

### 3.3. Chemiluminescence Assays

**Figure 11:**
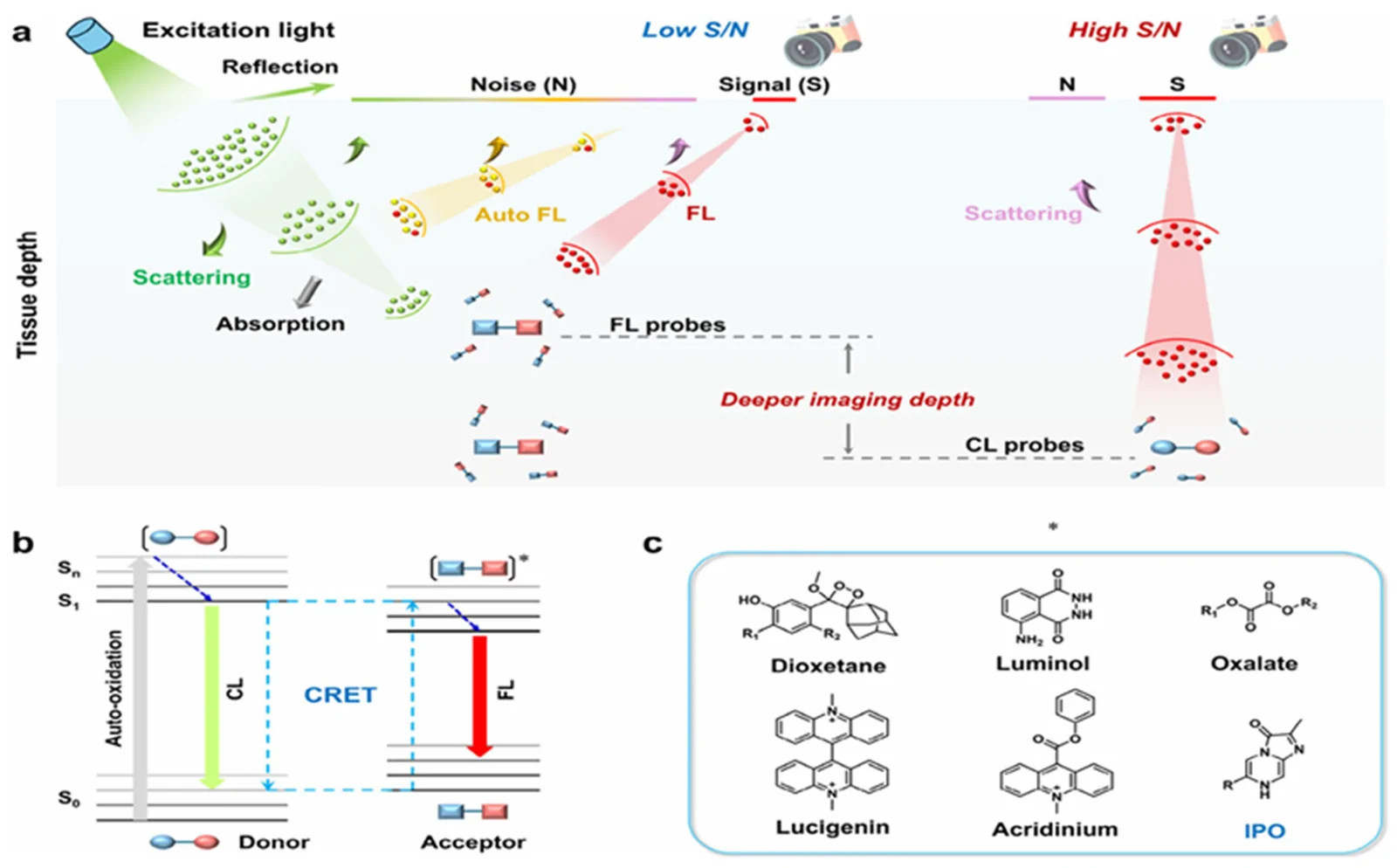
(**a**) Schematic illustration of fluorescence (FL) imaging and chemiluminescence (CL) light imaging and light attenuation in tissues. (**b**) Schematic illustrations of chemiluminescence resonance energy transfer (CRET). (**c**) Chemical structures of the commonly used chemiluminescent skeletons. Reprinted with permission from ref. [98].

Chemiluminescence refers to the light emission generated by a specific chemical reaction or an electron transition from the excited state back to the ground state. This technique is an effective tool for detecting biomarkers with the advantages of a free external light source, wide linear range, and high precision. It also has the characteristics of high automation, high throughput, rapid response, and low cost. Chemiluminescence probes can be designed to emit light at different wavelengths for imaging of Aβ species by tailoring their molecular structures and groups. The main groups of chemiluminescence probes are dioxetane, luminol, oxalate, lucigenin, and acridinium derivatives, which have been well summarized in previous reviews (Figure 11) [98,99]. The clinical translation of chemiluminescence imaging can be conducted with the instruments and workflows that are already available for fluorescence imaging in clinical practice. Nonetheless, the application of chemiluminescence imaging in neuroscience remains limited compared to fluorescence, primarily because its luminescence relies on specific chemical reactions often involving reactive oxygen species, and most of the chemiluminescence scaffolds have inherent limitations such as poor brain penetration, limited wavelength tunability, and insufficient stability.

**Figure 12:**
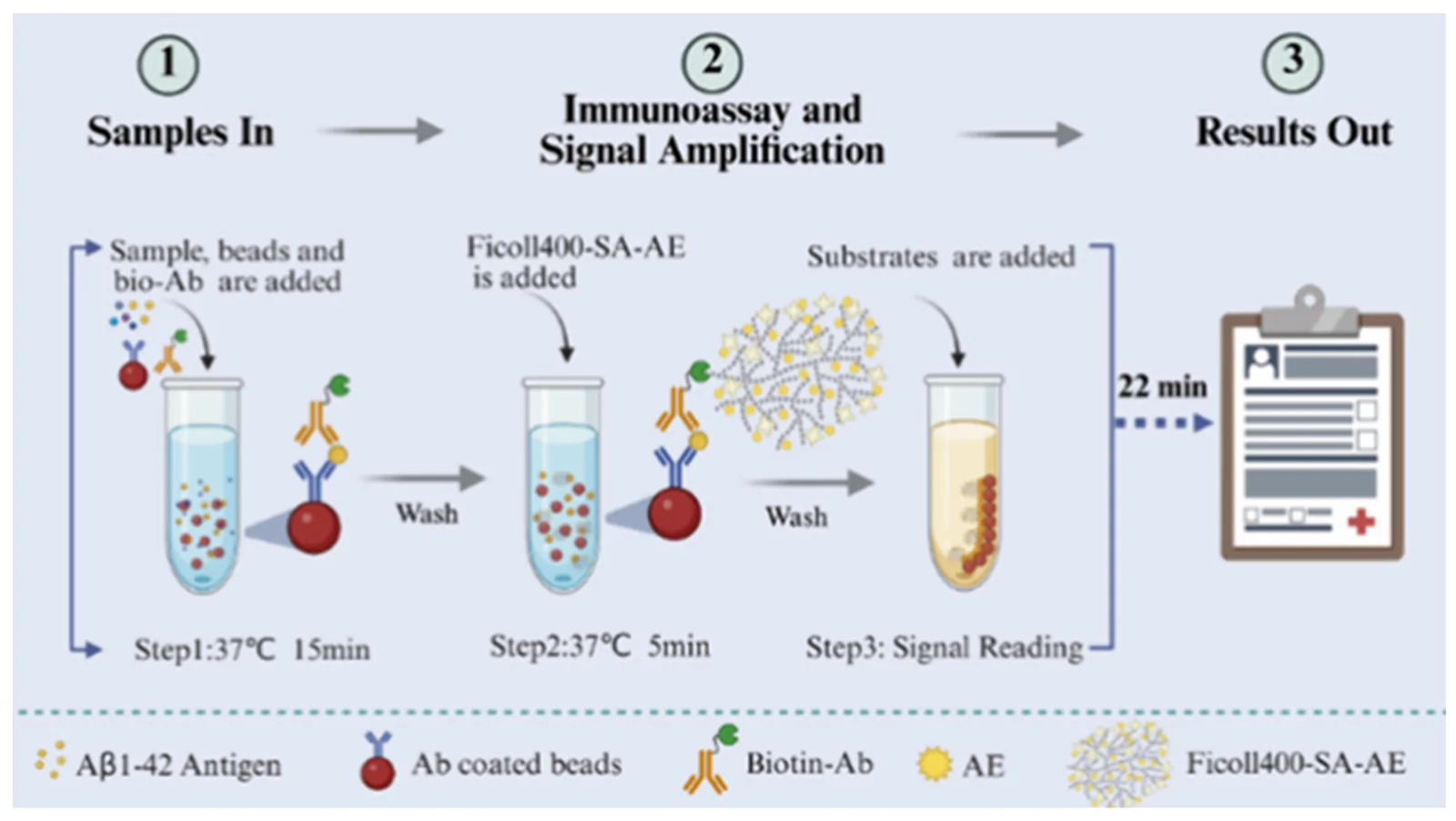
Schematic illustration of the methods and technical principles for ultrasensitive detection of Aβ1-42 in plasma based on Ficoll400-streptavidin macromolecular amplification carriers. Reprinted with permission from ref. [105].

Chemiluminescence immunoassay can provide a more sensitive process compared to ELISA. It is particularly suitable for detecting multiple protein biomarkers with concentrations exceeding 50 pg/mL. Although there are a series of commercially available chemiluminescence detection kits for AD-related protein biomarkers, including Aβ40, Aβ42, p-Tau, neurofilament light chain (NfL), and glial fibrillary acidic protein (GFAP), clinical evaluation data showed that their detection positive rates are lower than those of clinical diagnosis or Aβ-PET/Tau-PET [100,101]. The main reason may be insufficient detection sensitivity, making it difficult to distinguish subtle concentration changes under pathological conditions. In addition, a high proportion of mixed AD patients in the reference population may affect detection specificity. For this view, researchers have developed various signal amplification strategies to improve the sensitivity of chemiluminescence immunoassays such as enzymes [102,103], rolling circle amplification [104], and signal molecule-loaded carriers. Typically, Guo and colleagues designed an enhanced chemiluminescence immunoassay by combining acridinium ester with Ficoll400-streptavidin macromolecular amplification carriers for quantitative analysis of plasma Aβ1-42 (Figure 12) [105]. The optimized system delivered a 35-fold increase in luminescence intensity with a detection limit of 0.15 pg/mL and a quantitation limit of 0.31 pg/mL. The method exhibited excellent specificity, precision, and stability. The results for the assays of actual plasma samples were highly consistent with those achieved by commercial clinical kits. As an automated analysis method, the performance of this biosensor is superior to many traditional detection methods, making it very suitable for clinical detection of AD biomarkers.

### 3.4. SPR Biosensors

**Figure 13:**
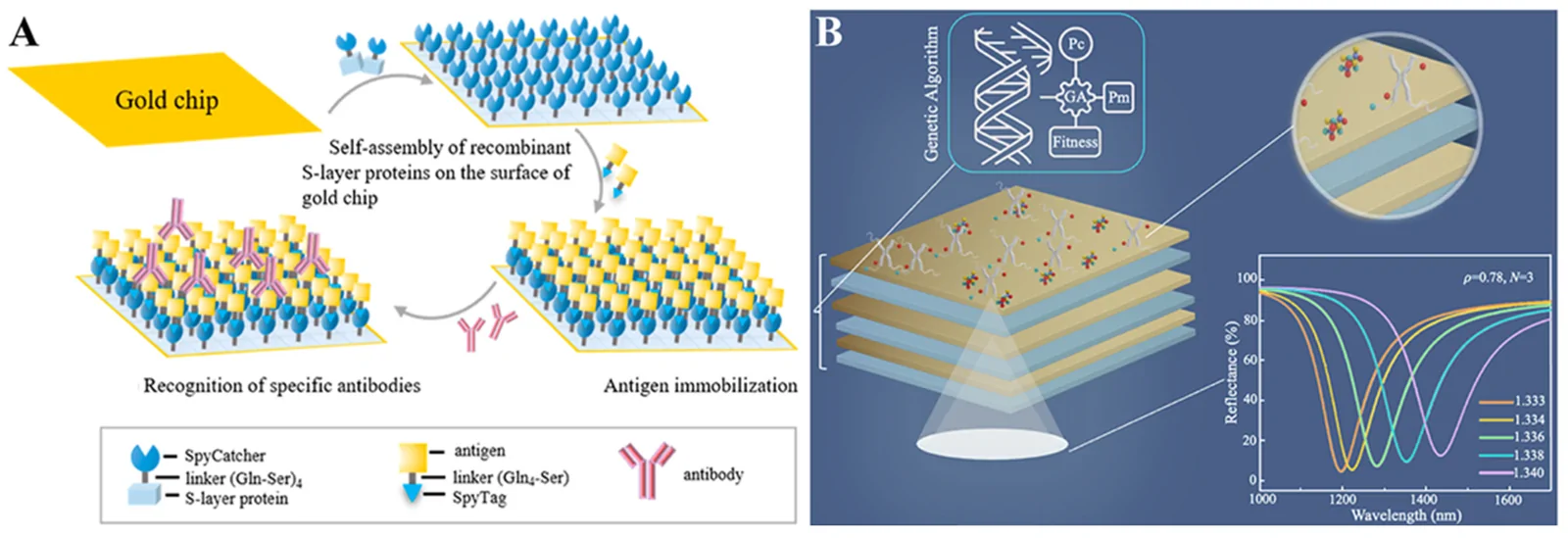
(**A**) Schematic illustration of the SPR nanoarray based on the artificial S-layer to detect serum or antibody on a gold chip. Reprinted with permission from ref. [109]. (**B**) Schematic illustration of the HMMs-SPR sensor. Inset: focused ion beam scanning electron microscope cross-sectional image of the HMMs-SPR composed of Ag/Al_2_O_3_ with ρ = 0.78, N = 3. Reprinted with permission from ref. [110].

SPR is one of the most important optical sensing techniques, which has been widely applied in various fields. It can typically monitor the analyte-triggered refractive index changes on a gold-coated sensor surface. The technique is highly sensitive to identify molecular interactions with a detection range covering a long gold film region (approximately 200 nm) [106]. SPR biosensors show the characteristics of low cost and high selectivity, and have the ability to perform on-site analysis. However, common AD protein biomarkers have molecular weights of several thousand to tens of thousands of Daltons, which will cause relatively small changes in the SPR refractive index through a direct detection model [107,108]. AuNPs-based SPR biosensors exhibit excellent optical performance, including enhanced signal amplification capability, good compatibility with organic compounds, and high oxidation resistance. The size and shape of AuNPs are critical to the performance of SPR biosensors. The intensity of the SPR signal is directly related to the size and morphology of AuNPs. For instance, AuNPs with a size smaller than 2 nm are unsuitable for fabricating SPR biosensors, as specific wavelengths of light may interfere with the electron oscillations on the surface of nanoparticles. Tang et al. proposed a mild biological immobilization strategy for biomarker detection by combining SpyTag-SpyCatcher interactions with bacterial S-layer proteins to construct ordered two-dimensional protein nanoarrays on gold sensing surfaces (Figure 13A) [109]. The immobilization method can preserve the natural conformation and biological activity of immobilized proteins, overcoming the defects of protein denaturation and disordered arrangement caused by traditional chemical cross-linking. SPR results demonstrated that the approach boosted detection sensitivity by eight times compared with chemical cross-linking, and it worked well for proteins unstable under chemical reaction conditions. The practicality of the nanoarrays was verified via serological detection of swine fever serum samples. This method can be extended to construct biosensing interfaces for developing various gold-based immunosensors. In addition, in order to improve the sensitivity of direct detection, Zhao and colleagues designed an SPR biosensor based on Ag/Al_2_O_3_ hyperbolic metamaterials (HMMs) (Figure 13B) [110], in which a genetic algorithm was adopted to globally optimize multiple structural parameters. Benefiting from the bulk plasmon polariton mode of HMMs, the biosensor produced intense local electric field enhancement, reaching a refractive index sensitivity of 43.62 μm/RIU, one order of magnitude higher than traditional SPR devices. The detection limit of this method was 0.18 pg/mL for Aβ42. Moreover, the GA-optimized HMM-SPR biosensor performed well in serum matrix, exhibiting outstanding sensing performance and good anti-interference capability and showing application potential for highly sensitive detection and screening of AD biomarkers.

**Figure 14:**
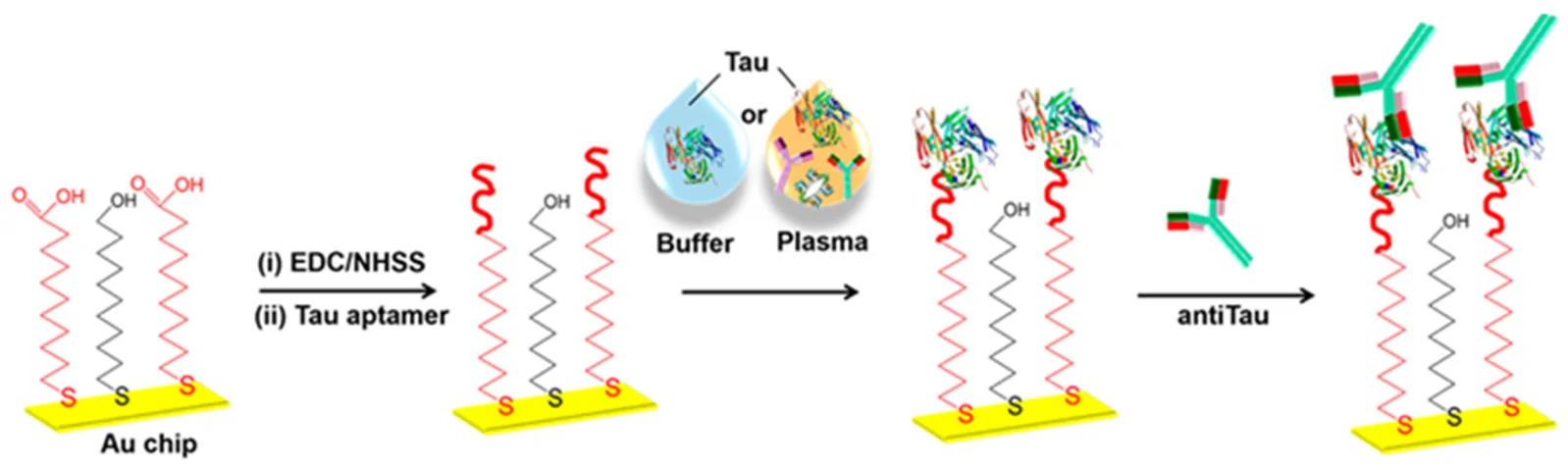
Schematic illustration of SPR strategy for Tau protein detection based on the signal amplification of antibody. Reprinted with permission from ref. [116].

In the sandwich detection model, SPR signals can be amplified by nanomaterials and large biomolecules due to their unique optical property and large bulk [111,112,113,114]. For example, we found that antibodies can be used as the SPR labels to achieve signal-amplified detection of Aβ40 and Aβ42 [114], as the molecular weight of the antibody (~150 kDa) is approximately 35 times higher than that of the two Aβ monomers. Kim et al. reported two sandwich SPR biosensors based on aptamer/antibody recognition systems for the detection of alpha-1 antitrypsin and Tau proteins [115,116]. For the detection of Tau, an anti-Tau antibody was used to recognize the target, specifically captured by the DNA aptamer modified on the gold chip (Figure 14). The method successfully measured Tau in serum samples from AD patients with a detection limit of 50 fM. In this work, the aptamer was immobilized on the gold chip modified with mercaptoundecanoic acid (MUA) and thiolated polyethylene glycol (PEG-SH) via acylation, and an antibody was used as the signal amplifier. In addition, researchers have developed highly tilted fiber Bragg grating (TFBG)-SPR sensing platforms integrated with multi-channel microfluidics, enabling simultaneous and sensitive detection of multiple AD biomarkers [117]. Different forms of Aβ42 in CSF have been in situ determined and, in parallel, distinguished with the detection limit in the range of 30–170 pg/mL.

**Figure 15:**
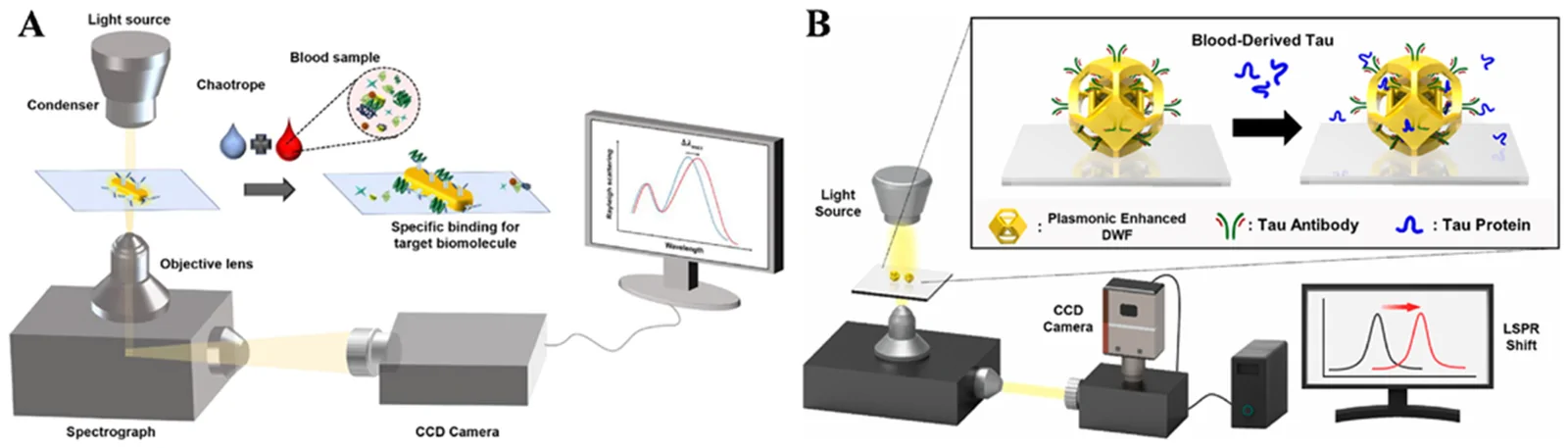
(**A**) Schematic illustration of nanoplasmonic biosensor system for delicate hemodiagnosis of AD. Reprinted with permission from ref. [119]. (**B**) Schematic illustration of the DWF-based single nanoparticle sensor for AD diagnosis by measuring LSPR shifts. Reprinted with permission from ref. [120].

LSPR is a type of SPR phenomenon characterized by the change in intensity and angle of reflected light varying with the properties of thin-film. Precious metal nanoparticles are widely used in LSPR technology because their surface can generate strong electromagnetic fields, enabling the detection of biomolecules at the nanoscale. Based on this principle, researchers have designed LSPR biosensors in which electrons in plasmonic nanoparticles are excited by light. The comparison of metal nanostructures of different sizes reveals that 200 nm structures offer higher sensitivity than 100 nm structures. This is because larger structures more effectively exploit refractive index changes to amplify signals and generate stronger local electromagnetic fields. Such intense near-field effects will enable the sensor to detect specific antigen–antibody binding with high sensitivity, largely unaffected by refractive index variations or non-specific interference in blood. Kim et al. developed a shape-encoded LSPR detection platform using spherical, short-rod, and long-rod AuNPs [118]. For the fabrication of LSPR chips, the suspensions of AuNPs were incubated on APTES-coated glass surfaces. PEG-modified AuNPs and nanorods were conjugated with anti-Aβ and anti-Tau monoclonal antibodies via EDC/NHS activation. The biosensor could detect Aβ40, Aβ42, and Tau in simulated blood with the detection limits of 34.9 fM, 26 fM, and 23.6 fM, respectively. In addition, guanidine hydrochloride was used as a chaotropic agent to overcome the limitations in blood-based LSPR detection (Figure 15A,B) [119,120]. The platform showed clinical potential for determining AD biomarkers in peripheral blood in view of its femtomolar detection limit, although the concentration of Tau is 10–100 times lower in blood than in CSF. By decreasing overlap in protein levels between age-matched controls and AD patients, the system diagnosed AD and assessed mild cognitive impairment progression.

Due to their excellent optical properties, silver-based nanomaterials have also been utilized for the design of LSPR platforms for specific sensing of AD-related protein biomarkers. For example, Haes and colleagues developed a LSPR biosensor using silver nanotriangles for the detection of Aβ-derived diffusible ligand (ADDL) associated with AD progression [121,122]. In this method, polystyrene microspheres are drop-coated onto a glass template to form a hexagonally close-packed monolayer. A thin chromium adhesion layer is then deposited over the microsphere monolayer. Silver is sputtered onto the surface, and the polystyrene nanospheres are removed by ultrasonic treatment, creating a silver nanotriangle array on the glass substrate. This LSPR platform was further used to screen ADDL and Tau protein in human brain extracts and CSF from AD patients [123].

**Figure 16:**
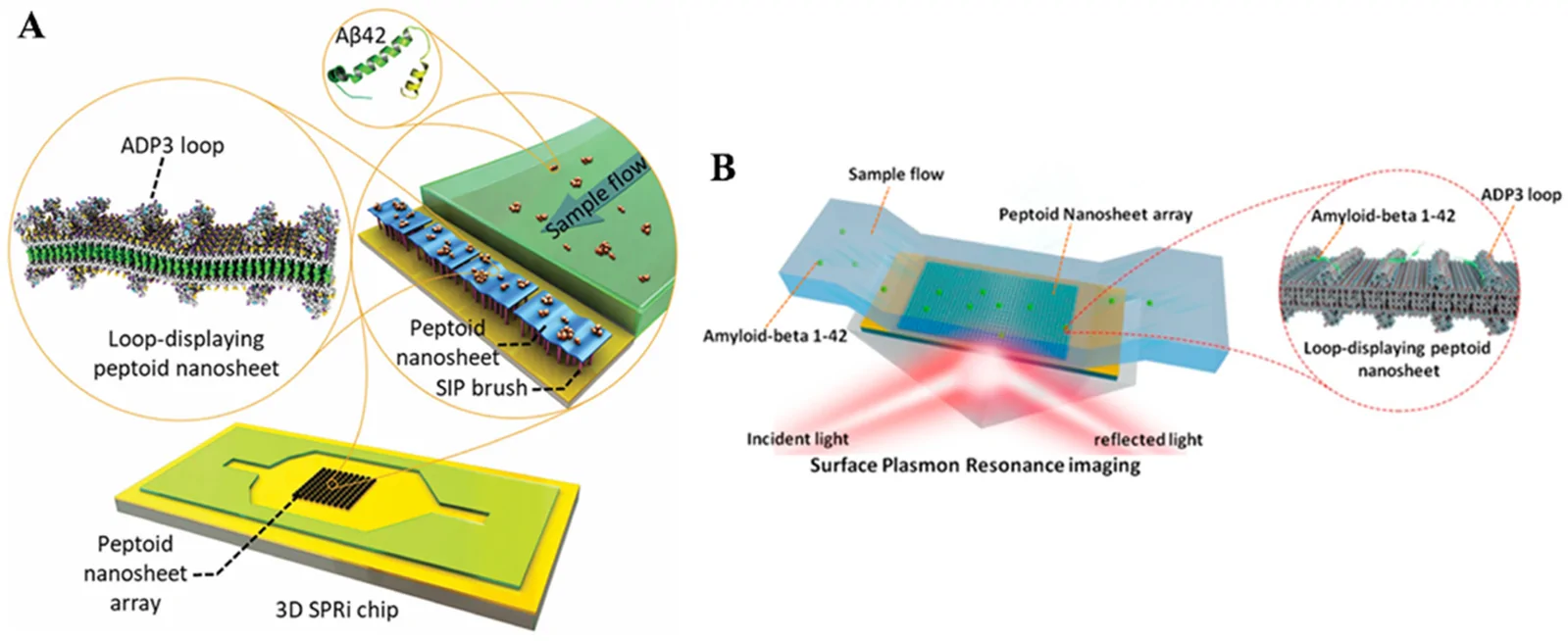
(**A**) Schematic illustration of the 3D SPRi sensor for the detection of AD serum in combination with loop-displaying peptoid nanosheets. Reprinted with permission from ref. [127]. (**B**) Schematic illustration of Aβ42 detection in the serum or plasma by combining the SPRi with loop-displaying peptoid nanosheets. Reprinted with permission from ref. [128].

SPR imaging (SPRi) biosensors, as the mainstream in situ label-free optical analytical instruments, have the characteristics of anti-interference capability, high sensitivity, high throughput, and fast response [124,125,126]. Leveraging the high surface activity, unique optoelectronic properties, good biocompatibility, ease of surface modification, miniaturization, and integration of nanomaterials, Zhu et al. developed a label-free biosensor for the diagnosis of AD using antibody-mimetic self-assembled peptide nanosheets with exposed Aβ42 recognition loops (Figure 16A) [127]. The nanosheets exhibited high affinity for serum Aβ42 and could identify AD serum with high sensitivity. The dense distribution of recognition loops on the peptide scaffold mimicked antibody structure and reduced non-specific binding in complex samples. This antibody-mimetic 2D material showed promising potential for blood-based AD diagnosis. In addition, Gao et al. developed a label-free blood Aβ42 quantification method based on the high-throughput SPRi biosensor with antibody-mimetic peptide nanosheets functionalized with Aβ42 recognition loops (Figure 16B) [128]. The system was used to specifically distinguish healthy individuals, AD patients, and subjects with amnestic mild cognitive impairment through sensitive recognition of Aβ42, providing a label-free, cost-effective, highly sensitive, and high-throughput blood test method for early screening of AD.

### 3.5. SERS Analysis

**Figure 17:**
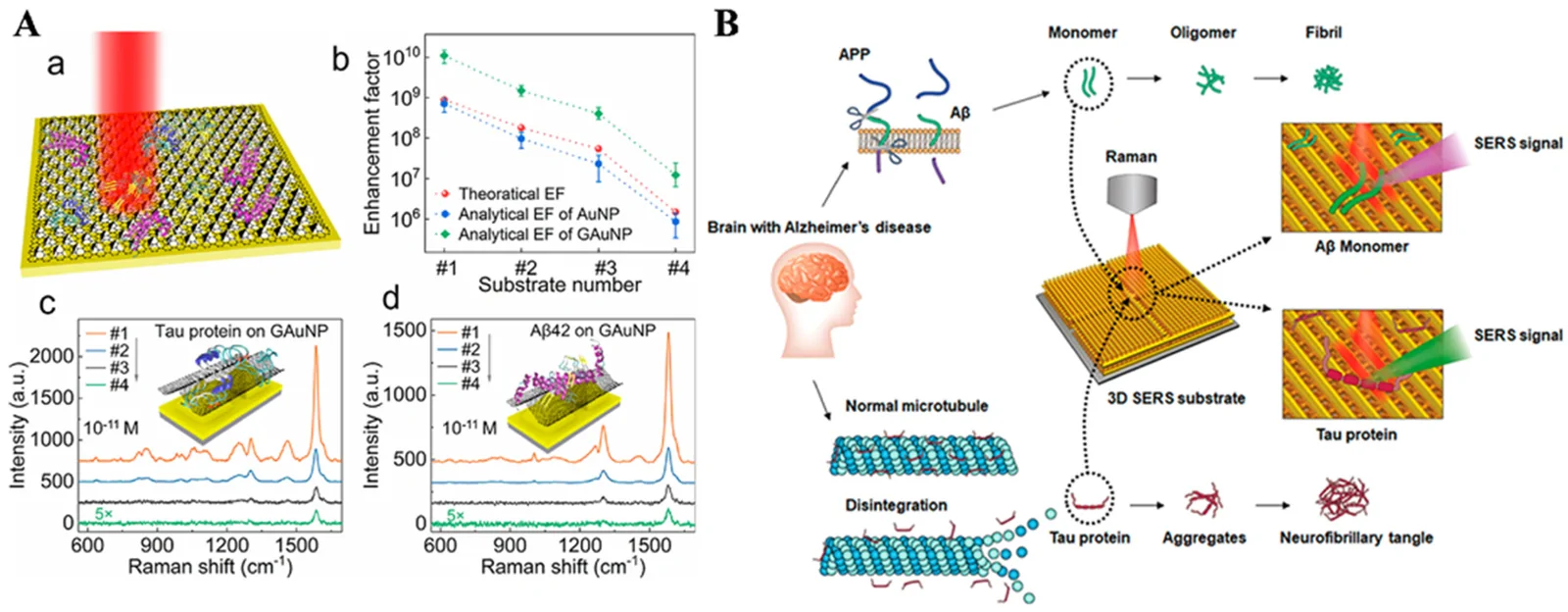
(**A**) Schematic illustration of SERS biosensor using the GAuNP substrate. (**a**) Schematic diagram of the detection principle. (**b**) Analytical SERS EFs of different substrates for the detection of R6G molecules. (**c**) SERS spectra of 10^−11^ M Tau protein obtained using GAuNP substrates. (**d**) SERS spectra of 10^−11^ M Aβ42 peptides. Reprinted with permission from ref. [141]. (**B**) Schematic illustration of label-free analysis of AD biomarkers using a carboxylic acid-functionalized, graphitic layer-coated three-dimensional SERS substrate. Reprinted with permission from ref. [150].

As a highly sensitive, label-free molecular recognition technique, SERS has attracted growing attention in the biosensing field, including the detection of AD biomarkers [129,130,131,132,133,134,135,136,137,138]. This technique, relying on the inelastic light scattering of monochromatic laser light, can monitor subtle structural changes in proteins, lipids, nucleic acids, and other biomolecules in plasma or serum. It can generate “molecular fingerprints” and has shown promise for disease classification and subtyping. By leveraging the LSPR effect on metal nanostructures, particularly in “hot spots”, SERS can amplify the Raman signals by several orders of magnitude and achieve single-molecule detection. Its advantages include ultra-low detection limit, capability for multiplex biomarker analysis, and powerful signal amplification ability. The characteristic peaks of Aβ and Tau proteins have been directly distinguished by SERS on the substrates (Table 3), such as chiral bimetallic Pt@Au octapods and Pt@Au triangular nanorings [139,140], wafer-scale graphene-Au nanopyramid hybrid [141], gold nanocubes [142], AuNPs-decorated polyvinyl alcohol microbubbles functionalized with copper ions and 4-mercaptobenzoic acid [143], AgNPs-coated chitosan film and nanofibers [144,145], ZIF-67-supported AgNPs [146], rose petal-structured micro/nano hierarchical silver films and AgNPs [147,148], lotus seedpod array [149], graphitic nanolayer [150], and graphitic carbon nitride@MOF (g-C_3_N_4_@MOF) [151]. A typical example is that Wu et al. fabricated a pattern-optimized graphene-gold nanopyramid hybrid (GAuNP) substrate and evaluated its SERS enhancement performance through theoretical simulation and experimental validation (Figure 17A) [141]. The larger nanopyramid with a narrower inter-pyramid gap yielded a maximum enhancement factor of 1.1 × 10^10^, enabling ultrasensitive assays of total Tau or p-Tau and Aβ42 with the detection limits of 1 fM and 10 fM, respectively. Furthermore, variations in the Raman signal within the amide III region (1200–1300 cm^−1^) revealed a concentration-dependent secondary structural transition of Aβ42 from α-helix to β-sheet. This dual acquisition of structural and quantitative information facilitated the early and rapid diagnosis of AD. In addition, Part et al. reported a three-dimensional SERS substrate coated with carboxylic acid-functionalized and graphitic nanolayer for monitoring the conformational and concentration changes in Aβ and Tau proteins (Figure 17B) [150]. The substrate consisting of well-defined, uniform gold nanowire arrays was fabricated by sequential nano-transfer printing. It achieves an enhancement factor as high as 5.5 × 10^5^, the highest value reported to date, facilitating quantitative analysis of protein secondary structure changes. Based on the intensity of the main band at 1380 cm^−1^, the linear range for Aβ detection was 1–100 nM.

**Figure 18:**
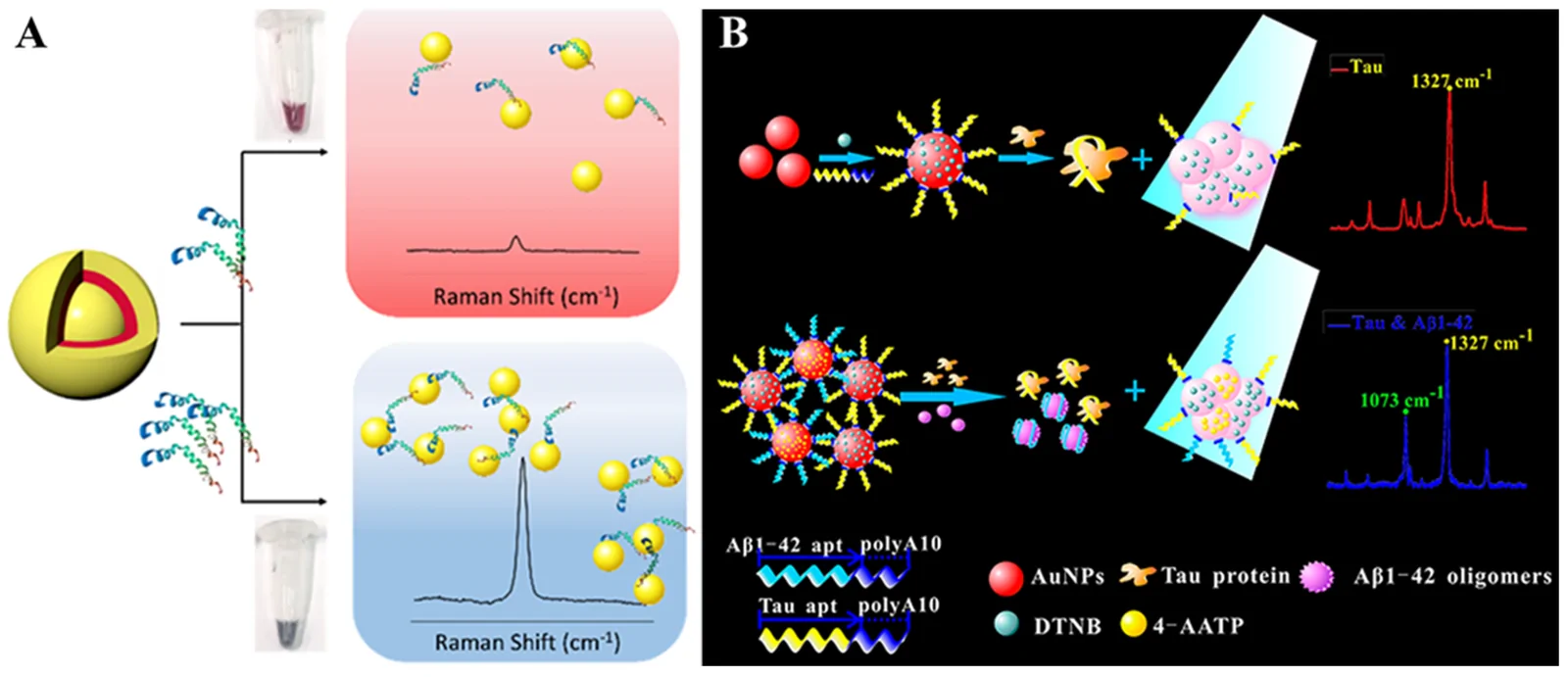
(**A**) Schematic illustration of Raman-SERS spectra acquired against Au@RR@AuNPs with different quantities of Aβ42 ranging from 0.04 to 105 ng/mL. Reprinted with permission from ref. [154]. (**B**) Schematic illustrations of PAapt-AuNPs conjugate-based SERS biosensor for sensitive detection of Tau protein and multiplexed SERS biosensor for simultaneous detection of Aβ(1-42) oligomers and Tau protein using different Raman dye-coded PAapt-AuNPs conjugates. Reprinted with permission from ref. [155].

Highly specific detection of AD biomarkers largely relies on the scientific design of recognition strategy. Modification of SERS substrates with target-specific biorecognition elements such as antibodies and aptamers can improve the detection sensitivity and specificity for AD biomarkers [152]. Khan et al. reported the label-free SERS analysis of Aβ with aptamer-modified mesoporous gold (mesoAu) and gold-coated magnetic nanoparticle (Au@MNP) [153]. The mesoAu substrate was prepared by electrodeposition with pore sizes ranging from 30 to 70 nm, and the Au@MNP was synthesized by a hydrothermal method. The gold substrate was modified with PEG and a DNA aptamer to form a self-assembled monolayer for target recognition in complex media. Based on the intrinsic SERS fingerprints of phenylalanine, the method is capable of determining Aβ42 in CSF with a detection limit of 0.15 pM within 20 min. The clinical validity was demonstrated by testing 30 CSF samples of AD, mild cognitive impairment, and control. Dallari et al. designed multilayer bioorthogonal SERS nanoprobes (Au@RRs@AuNPs) by incorporating Raman reporter molecules between two layers of AuNPs for highly sensitive detection of Aβ42 (Figure 18A) [154]. The nanoprobes exploited the high affinity between Aβ42 and the gold surface, promoting Cu^2+^-mediated aggregation of AuNPs. The aggregation degree was proportional to the concentration of Aβ, thereby enabling quantitative analysis based on the change in the SERS signal intensity. The method successfully detected Aβ42 at the pg/mL level in clinical CSF samples with a detection limit of 0.65 ng/mL, while exhibiting no cross-reactivity with Aβ40. Zhang et al. developed a universal multiplex SERS platform based on polyadenine (polyA) aptamer-AuNP (PAapt-AuNP) conjugates for simultaneous detection of Aβ1-42 oligomers and total Tau (Figure 18B) [155]. Target binding triggered the detachment of aptamers and the aggregation of AuNPs, generating strong SERS signals from different Raman dyes for dual recognition. The system exhibited outstanding salt tolerance and high specificity with the detection limits of 4.2 × 10^−4^ pM for Tau and 3.7 × 10^−2^ nM for Aβ1-42 oligomers. The assay can be completed within 15 min. Verified with artificial CSF, this simple and efficient multiplex sensing tool holds promise for auxiliary diagnosis of AD by monitoring the related biomarkers.

**Figure 19:**
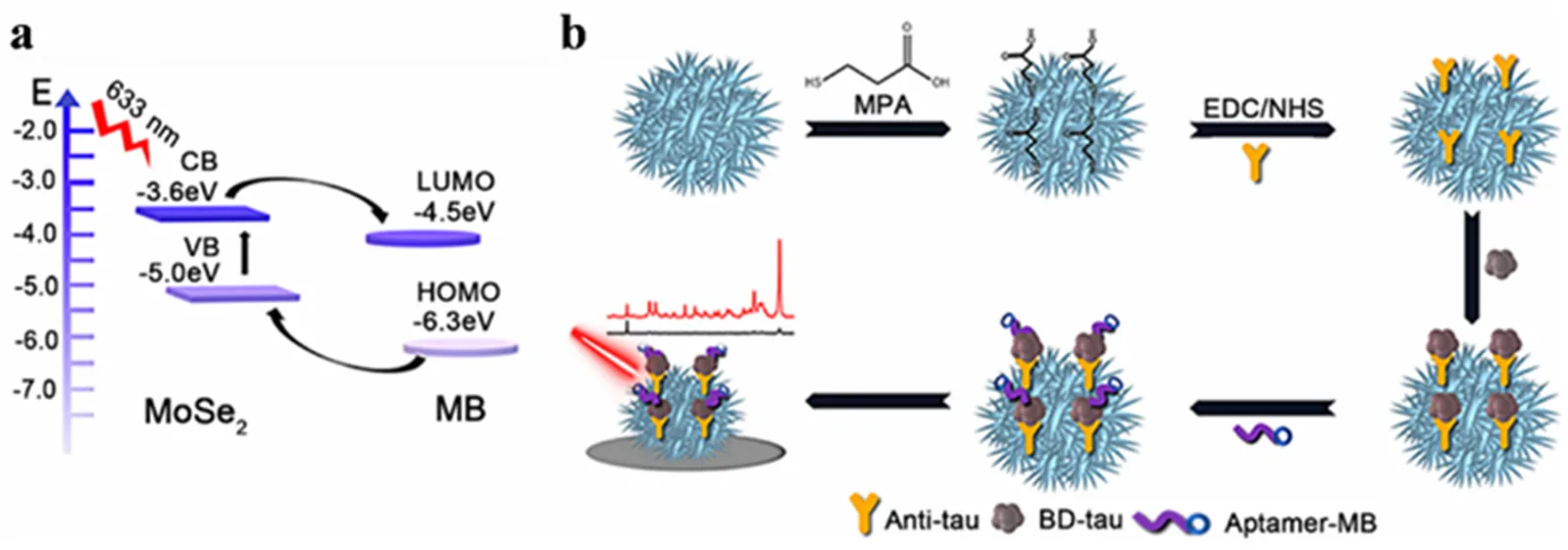
(**a**) Schematic illustration of mechanism for charge transfer of MB and MoSe_2_. (**b**) Construction of SERS substrate based on MoSe_2_ microspheres for determination of BD-tau. Reprinted with permission from ref. [158].

Target capture and signal transduction can be achieved through a classical sandwich immunoreaction strategy using enzymes or SERS nanoprobes as the signal labels [156,157]. For example, Zhou et al. fabricated urchin-like MoSe_2_ microspheres through a one-step hydrothermal method and constructed a SERS detection platform for brain-derived Tau (BD-Tau) (Figure 19) [158]. The method had a high SERS enhancement factor (1.44 × 10^7^) due to charge transfer effect, molecular resonance, and porous structure-induced enrichment. Through the aptamer-antibody sandwich detection method, the biosensor had a linear range of 0.05–200 pM and a detection limit as low as 0.028 pM with strong selectivity against various interfering substances. Animal plasma experiments indicated that the platform could differentiate AD model mice from other neurodegenerative disease mice, offering a specific and convenient tool for the differential diagnosis of AD.

**Figure 20:**
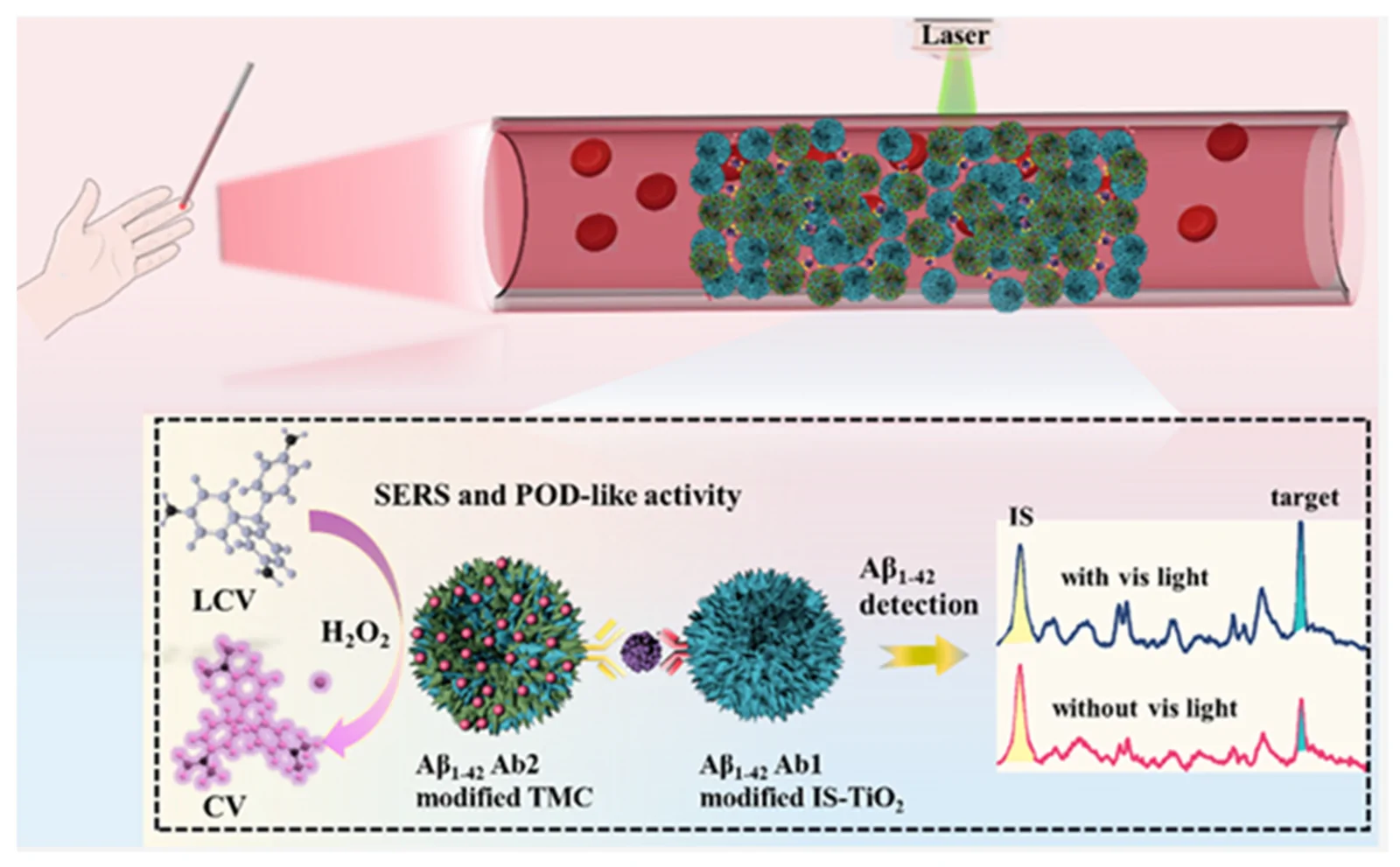
Schematic illustration of hierarchical tandem heterojunction with visible light enhanced peroxidase-like activity for all-in-capillary self-calibrating SERS immunoassay of Aβ1-42. Reprinted with permission from ref. [160].

Researchers have further refined nanoprobe-based SERS systems to improve detection selectivity and sensitivity, in which nanoprobes are usually labeled with Raman reporters and conjugated with antibodies or aptamers, enabling highly specific molecular recognition [159]. The nanotags can not only significantly enhance the SERS signal intensity but also support multiplex detection, making it ideal for measuring trace levels of AD biomarkers in blood samples. For example, Chen et al. developed an all-capillary SERS analytical system integrated with visible-light-enhanced nanozymes for sensitive and convenient detection of AD biomarkers (Figure 20) [160]. A hierarchically tandem heterojunction (TiO_2_/MoS_2_/CFe_2_O_4_) was employed as a peroxidase-mimicking nanozyme, which greatly promoted the separation and transfer of photogenerated charges under visible light. This nanozyme catalyzed the oxidation of colorless crystal violet, producing a characteristic Raman signal at 923 cm^−1^. To ensure analytical reliability, the inner capillary wall was modified with TiO_2_ as an internal standard, enabling signal self-calibration. This integrated platform allowed the direct collection of finger-prick blood without pretreatment for full-capillary SERS detection. It achieved ultrasensitive detection of Aβ1-42, with a linear range of 0.1–1000 pg/mL, a detection limit of 89 ng/mL, and 94.3–101% recoveries in human serum. The method has been successfully applied to distinguish clinical serum samples from AD patients and various control groups, revealing statistically significant differences in Aβ42 levels.

**Figure 21:**
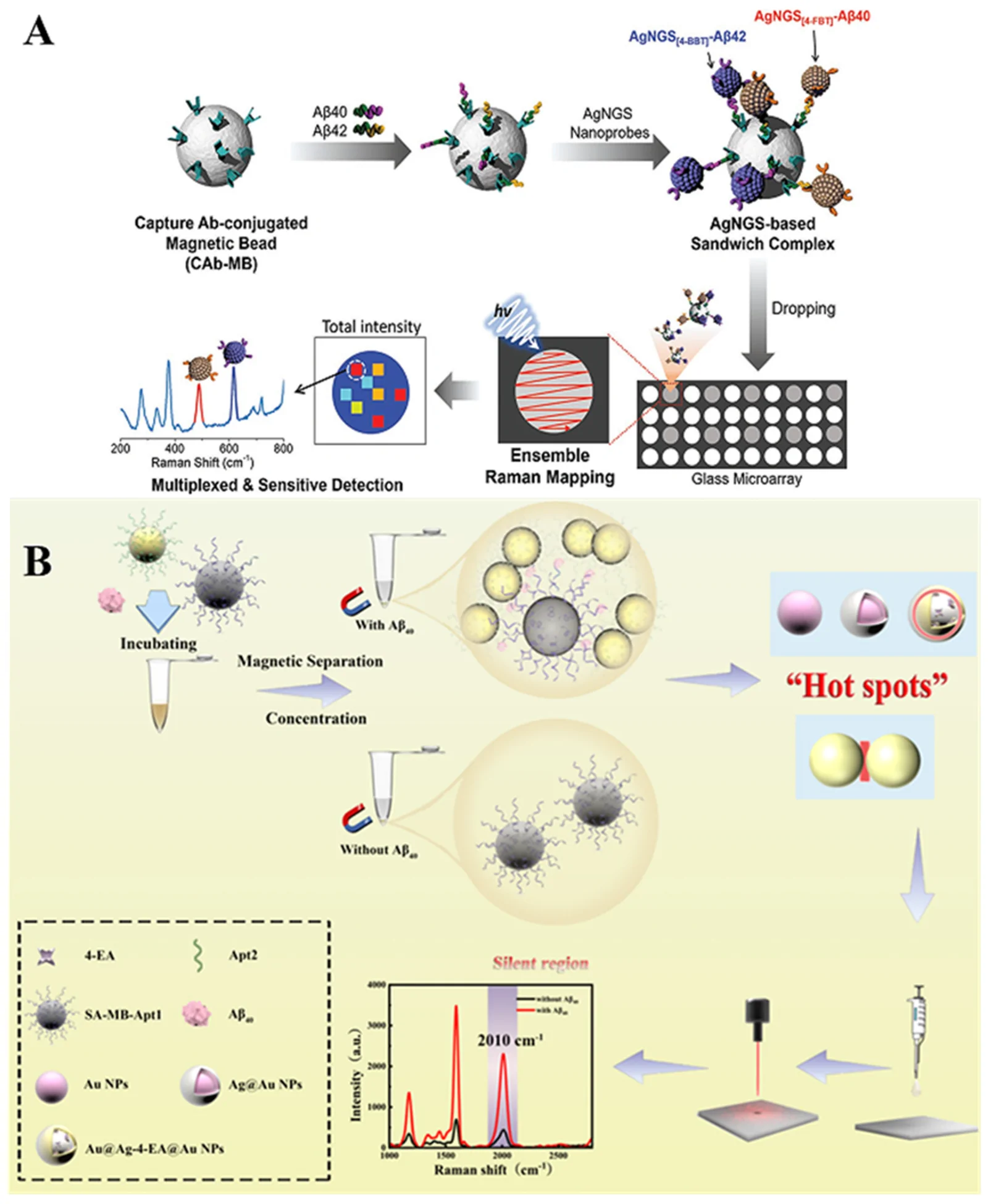
(**A**) Schematic illustration for the multiplexed detection of Aβ40 and Aβ42 over Aβ antibody-conjugated AgNGS nanoprobes in a sandwich immunoassay. Reprinted with permission from ref. [162]. (**B**) Schematic illustration for the preparation of Au@Ag-4-EA@Au NPs and SERS-based sandwich aptasensor for Aβ40 detection. Reprinted with permission from ref. [164].

A major obstacle in SERS-based detection of AD biomarkers is the interference from abundant blood proteins and other matrix components. In parallel, the performance of SERS biosensors is intimately linked to substrate design, as enhancement factors and detection limits are directly related to the substrate capacity for generating intense local electromagnetic fields and enabling robust surface–analyte interactions [161,162,163,164,165]. Luo et al. developed a SERS-based immunosensing platform for Aβ detection with Raman reporter-functionalized flower-like MoS_2_ immunoprobes [161]. Ag nanocube (Ag NC)-decorated polyvinylidene fluoride (PVDF) films were used as the SERS substrates. The SERS activity of the resulting sandwich immunocomplexes was robustly enhanced by triggering the piezoelectric effect of the PVDF matrix under proper pressure, enabling ultralow detection limits of 0.391 and 0.156 pg/mL for Aβ40 and Aβ42, respectively. Yang’s group developed a facile wet synthesis method to prepare silver nanogap shell (AgNGS) SERS nanoprobes, in which the reaction kinetics were regulated by the labeled Raman molecules to precisely control nanogap size at the nanometer scale (Figure 21A) [162]. The as-prepared AgNGS exhibited high SERS enhancement efficiency and long-term signal stability, and different Raman tags produced non-overlapping spectral signals for multiplex analysis. Combined with sandwich immunoassays, the AgNGS-based nanoprobes could realize simultaneous detection of different Aβ species in human serum with negligible cross-reactivity. The detection limits were found to be 0.25 pg/mL and 0.33 pg/mL for Aβ40 and Aβ42, respectively. The linear detection range was two orders of magnitude wider than conventional ELISA. This nanogap SERS probe is a promising alternative for highly sensitive and multiplex detection of AD biomarkers in clinical blood samples. Hong et al. reported a novel background-free SERS aptasensor that integrated dual-aptamer recognition and magnetic enrichment for the detection of Aβ40 (Figure 21B) [164]. The biosensor adopted Au@Ag-4-EA@Au core–shell nanoparticles and Raman-silent regions to eliminate biological background interference and generate abundant plasmonic hot spots for signal amplification. This platform achieved a wide linear range from 0.1 pM to 10 nM and a detection limit of 25 fM, with excellent uniformity, reproducibility, and storage stability. Validated by clinical serum samples, the biosensor showed high consistency with commercial ELISA kits and effectively distinguished AD patients from healthy individuals. This background-free SERS strategy provided a reliable solution for early clinical diagnosis of AD.

Lateral flow immunoassay (LFIA) is widely used as a rapid point-of-care testing technique in clinical diagnosis on account of its accessible, fast, and low-cost characteristics. The integration of SERS with LFIA and microfluidics can improve the detection performance of physical enrichment and signal acquisition [166,167,168,169,170,171]. Zhang et al. fabricated a dual-signal LFIA strip for simultaneous colorimetric and SERS detection of AβO and phosphorylated Tau (p-Tau^396,404^) in plasma [167]. Adopting a double-antibody sandwich structure, the two targets were synchronously captured by the strip, and the colorimetric/SERS nanotags were attached for dual-signal readout, effectively reducing positive and negative false results from single-signal detection. The detection limit was 3.8 pg/mL for p-Tau^396,404^ with an area under the curve (AUC) value of 0.933 in differentiating AD patients from healthy elderly individuals. This platform only required one drop of plasma and did not require bulky instruments to deliver the results within 25 min, facilitating its application in communities and primary healthcare facilities. After clinical validation, this dual-signal LFIA strip is expected to become a low-cost auxiliary tool for AD diagnosis.

**Figure 22:**
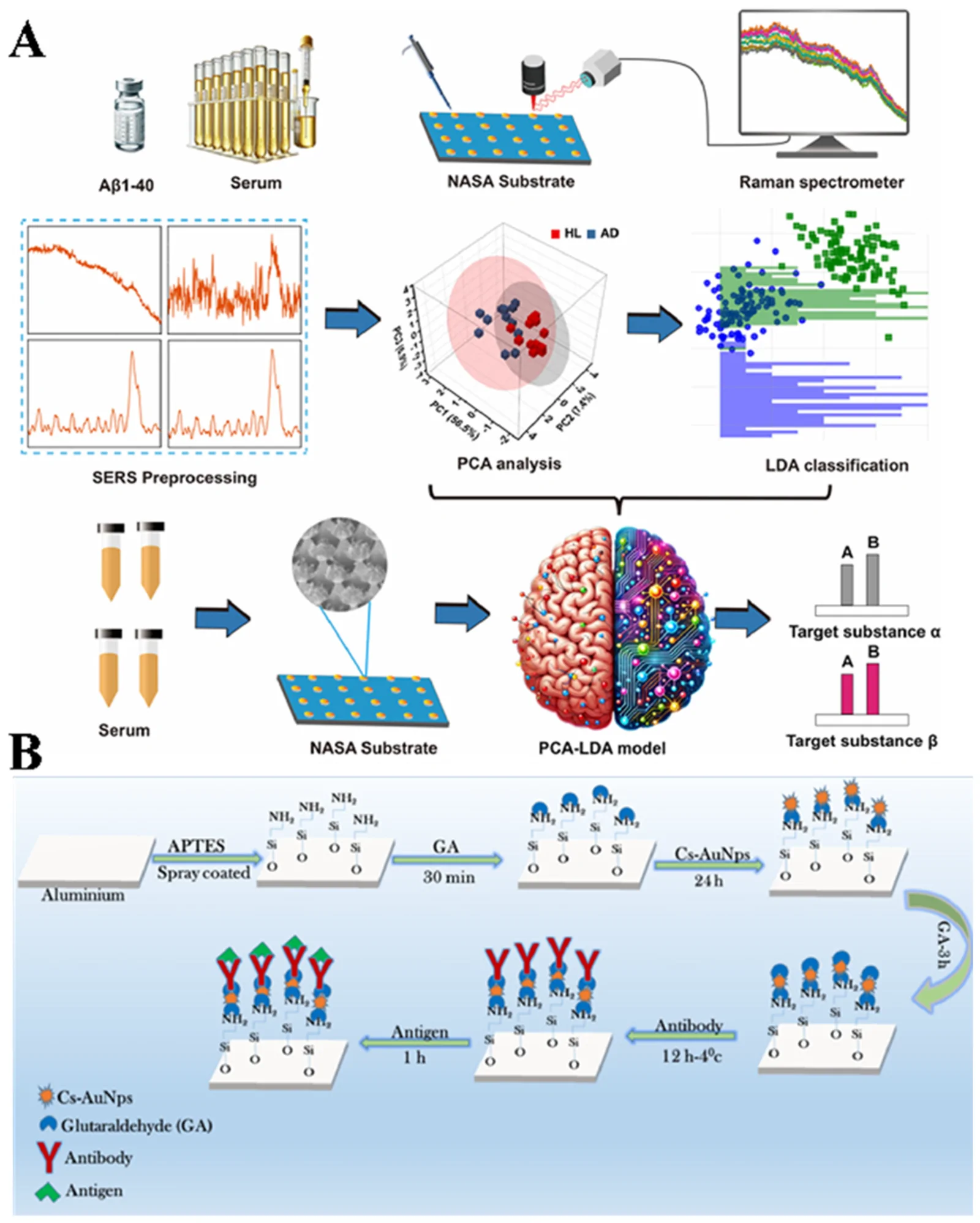
(**A**) Schematic illustration of a serum analysis platform, combining machine learning and SERS techniques for identifying AD rats. Reprinted with permission from ref. [173]. (**B**) Schematic illustration of preparation and application of an Al-SERS-based immunoassay platform for the detection of Aβ40, Aβ42, p-Tau and total Tau. Reprinted with permission from ref. [174].

The combination of biosensors with AI can systematically enhance detection performance and diagnostic efficiency. The integration of AI into SERS-based detection has primarily facilitated automated denoising, baseline correction, and feature extraction from complex SERS spectra [172]. By reducing subjective errors associated with manual interpretation, AI integration markedly elevates the sensitivity, accuracy, and overall robustness of the detection system. Lu et al. constructed a label-free serum analysis platform combining SERS with a principal component analysis-linear discriminant analysis (PCA-LDA) machine learning algorithm for high-accuracy identification of AD in rat models (Figure 22A) [173]. The SERS substrates fabricated with highly ordered nanostar arrays (NASA) structures showed excellent uniformity, good reproducibility, and high stability. The platform could sensitively monitor the signal change induced by 10 pM target protein. Through multivariate analysis of SERS spectra acquired from AD and control rat serums, the PCA-LDA model achieved 95.8% accuracy, 100% sensitivity, and 92% specificity in distinguishing the two groups with 0.97 AUC. This method not only enabled early non-invasive identification of AD but also revealed molecular-level alterations in proteins, lipids, and nucleic acids associated with the disease, thereby offering insights for elucidating AD pathogenesis. Resmi et al. developed an AI-SERS immunoassay platform to distinguish patients with mild cognitive impairment from those with AD (Figure 22B) [174]. Using chitosan-coated AuNPs probes, they successfully obtained sensitive SERS signals for blood biomarkers such as Aβ42 and Tau. When classifying SERS data with the support vector machine (SVM) algorithm, the sensitivity for distinguishing mild cognitive impairment from AD ranged from 58% to 100%, with specificity between 40% and 54%. Despite a series of novel works having been reported, machine learning models trained on small or homogeneous datasets are prone to overfitting, sensitive to spectral preprocessing choices, and susceptible to batch effects and inadequate experimental procedures. Therefore, the development of AI-assisted SERS methods should incorporate external validation, locked algorithms, standardized preprocessing pipelines, and clinically representative datasets. Previous reviews have systematically discussed these validation requirements for AI-assisted SERS spectroscopic analysis, and interested readers can consult the original references for a comprehensive discussion [175,176].

**Table 3:** Performances of SERS platforms for the detection of Aβ and Tau.

Method	Material	Target	Detection Limit	Linear Range	Sample	Ref.
Label-free	MoSe_2_	Tau	0.028 pM	0.05–200 pM	Brain tissues	[137]
	Ab-DNA/MB/Ag	AβO	4.6 fM	0.01–10^3^ nM	Serum (3 HI + 3 AD)	[138]
	L/D-Pt@Au octapods	Aβ40, Aβ42	0.23 pg/mL, 0.19 pg/mL	1–10^4^ pg/mL	CSF (3 HI + 3 AD)	[139]
	L/D-Pt@Au TNRs	Aβ42	0.045 pM	10 fM–1 nM	CSF (1 HI + 5 AD)	[140]
Sandwich	SDS-PAGE/Au/FDTD	Tau	3.21 fM	10–10^12^ fM	Plasma (2 HI + 5 AD)	[156]
	4-NBT-BCS, TFMBA/4-MBA	Aβ40, Aβ42	0.23 fM, 19 aM	0.01–10 pM	Spiked human plasma	[157]
	AuNPs + AuNWs	Aβ40	70 pM	100–740 pM	Mice plasma (3 HI + 3 AD)	[158]
	Au@MNPs/PEG/DNA	Aβ42	0.15 pM	0.01–100 ng/mL	CSF (10 HI + 10 AD)	[159]
	TiO_2_/MoS_2_/CoFe_2_O_4_	Aβ42	0.089 pg/mL	0.1–10^3^ pg/mL	Spiked human serum	[160]
	PVDF-AgNCs/MoS_2_	Aβ40, Aβ42	0.391 pg/mL, 0.156 pg/mL	1–10^7^ pg/mL	Real human CSF	[161]
	AgNGSs/Si NPs	Aβ40, Aβ42	0.25 pg/mL	Not reported	Spiked human serum	[162]
	Ag/4-MBA + Fe_3_O_4_@GOs	Aβ42, p-Tau-181	1.62 fg/mL, 5.74 fg/mL	10–10^5^ ng/mL; 0.001–10^2^ pg/mL	Serum (22 HI + 21 AD)	[163]
	SA-MBs-Apt1/Apt2-Au@Ag-4-EA@AuNPs	Aβ40	25 fM	0.1–10^4^ pM	Serum (3 HI + 3 AD)	[164]
LFIA	AuNPs/NBA/4-ATP	Aβ42, p-Tau181	0.1 pg/mL	0.1–10^4^ pg/mL	Spiked human serum	[166]
	4-MBA or DTNB@AuNPs	Aβ42O, p-Tau^396,404^	0.3 pg/mL, 1 pg/mL	0.3–6.4 × 10^3^ pg/mL, 1–6.4 × 10^4^ pg/mL	Plasma (15 AD + 15 NEI)	[167]
	AuSt@4-NTP@SiO_2_ NPs or AuSt@4-MBN@SiO_2_ NPs	Aβ42	12.1 pM	0.1–50 nM	Spiked human CSF	[168]
	UACPD	p-Tau^396,404^	1 fM	0.1–10^5^ fM	Blood (6 HI + 12 AD)	[169]
	Au/4-MBA@SiO_2_	Aβ42, Aβ40, Tau	138.1 fg/mL, 191.2 fg/mL, 257.1 fg/mL	1–10^6^ pg/mL	Phosphate buffer	[170]
	Au@SiO_2_	Aβ40, Aβ42	16.8 fg/mL, 15.3 fg/mL	0.1–10^5^ pg/mL	Phosphate buffer	[171]

Abbreviation: AuNC, Au nanocavity; AuNPs, gold nanospheres; DTNB, 5,5-dithiobis-(2-nitrobenzoic acid; Ab, antibody; MB, molecular beacon; L/D-Pt@Au TNRs, L/D-Pt@Au triangular nanorings; SDS, sodium dodecyl sulfate; PAGE, polyacrylamide gel electrophoresis; FDTD, finite-difference time-domain; 4-NBT-BCS, bumpy core–shell modified with 4-nitrobenzene-thiol; TFMBA, 2,3,5,6-tetraffuoro-4-mercaptobenzoic acid; 4-MBA, 4-mercaptobenzoic acid; AuNWs, walnut-like gold nanoparticles; PEG, polyethylene glycol; Au@MNPs, gold-coated magnetic nanoparticles; PVDF, polyvinylidene fluoride; AgNGSs, Ag nanogap shells; Si NPs, silica nanoparticles; Fe_3_O_4_@GOs, tannin-capped silver nanoparticles and magnetic graphene oxide; Apt1, first aptamer; Apt2, second aptamer; SA, streptavidin; MBs, magnetic beads; 4-EA, ethynylaniline; NBA, Nile rule A; 4-ATP, aminothiophenol; Au nanostar (AuSt); 4-nitrothiopheno (4-NTP); MBN, 4-mercaptobenzonitrile; UACPD, ultrasensitive antibody pair-based CRISPR protein detection; NEI, normal elderly individual.

### 3.6. Other Methods

**Figure 23:**
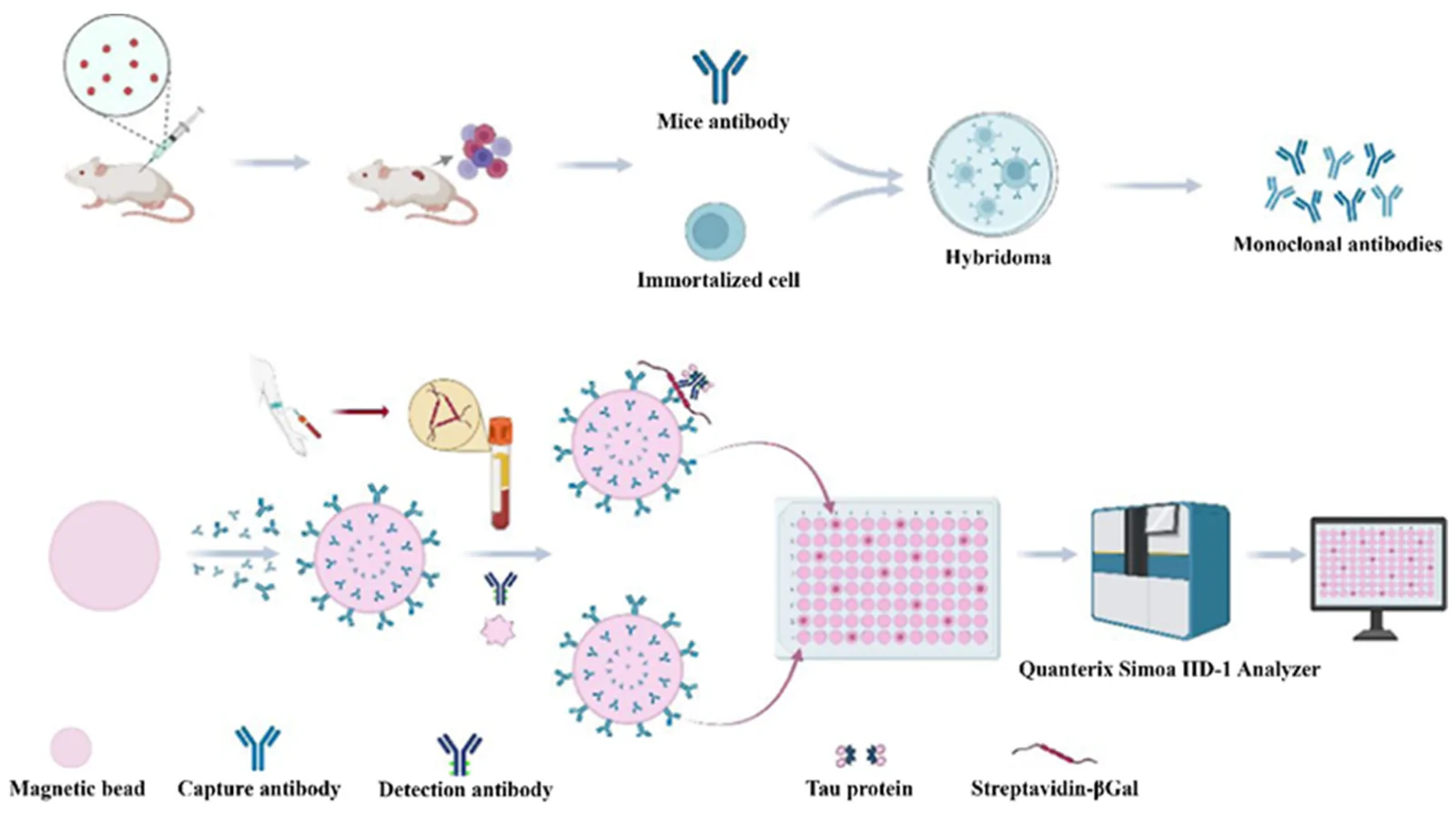
Schematic illustration for development of new blood immunoassays and biomarkers for the diagnosis of AD. Reprinted with permission from ref. [179].

Single-molecule immunoassays can be developed through direct imaging, scanning, and digital signal amplification. They can combine the magnetically assisted enzyme-linked immunoassays with microarray chips, enabling highly sensitive detection of individual protein biomarkers [177]. Integrating a highly sensitive fluorescence amplification system with digital counting in microarrays can boost detection sensitivity to fg/mL levels, which is 1000 times higher than conventional ELISA [178]. It meets the testing requirement for AD biomarkers in all disease stages and is especially suitable for early detection of plasma/serum biomarkers. Clinical investigation based on single-molecule immunoassays for AD biomarkers has confirmed their clinical applicability. Recently, Li et al. developed an immunoassay platform for distinguishing different forms of Tau proteins based on both ELISA and single-molecule immunoassay (Simoa) arrays (Figure 23) [179]. Tau fragments in human plasma were quantified, and the ratios of novel blood biomarkers were identified. Six monoclonal antibodies have been developed, supporting nine ELISA tests and three Simoa arrays. The detection limits for measuring three distinct Tau fragments in plasma by Simoa were 0.18 pg/mL, 0.12 pg/mL, and 2.70 pg/mL. The study first demonstrated the ratios of p-Tau217/Tau and Tau(1-22)-(187-208)/Tau(1-22)-(210-227) in blood. The platforms showed promising potential as effective tools for screening AD blood biomarkers. In addition, dried plasma spot testing with Simoa enables remote collection and quantitative analysis without the need for cold storage, refrigerated transport, or centrifugation. AD biomarkers in capillary blood-derived dried plasma spot, venous blood-derived dried plasma spot, and matched plasma have been measured by Simoa [180]. The results demonstrated that the technology holds potential for the detection of AD-related blood biomarkers, especially for screening in remote areas and primary healthcare settings. However, its performances such as sensitivity and precision and clinical suitability still need further validation.

## 4. Challenges in Biomarker Detection and Comparative Analysis of Technology Platforms

Despite the excellent analytical performance of the above-mentioned optical methods, the successful realization of their clinical translation still faces two core challenges. First, the concentrations of AD biomarkers in peripheral blood are extremely low due to the restrictive blood–brain barrier, and the distribution of different biomarkers in the population shows considerable overlap. For example, plasma Aβ42 is typically below 20 pg/mL, and p-Tau181 ranges from approximately 10 to 25 pg/mL. These concentrations are susceptible to biological factors such as age, renal function, comorbidities, and genetic background, making it difficult for any single biomarker to fully reflect the complex pathological processes of AD. Second, there are significant differences in the results from different laboratories, detection platforms, and methods, and the lack of standardized protocols and reference materials makes cross-center and cross-platform comparison and replication of results challenging. In addition, from a neurologist’s perspective, these challenges can be further categorized into three specific types of obstacles. First, pre-analysis variables can significantly affect the stability and reliability of detection results, including anticoagulant type, plasma versus serum selection, storage temperature, freeze–thaw cycles, hemolysis, renal function, age, comorbidities, and blood–brain barrier integrity [181]. Second, analytical variables, such as assay calibration, antibody or aptamer specificity, lot-to-lot variation, matrix effects, and multiplex interference, directly determine the analytical performance of biosensors in complex biological samples. Third, clinical variables, including disease stage, mixed pathology, and differential diagnosis requirements, further complicate clinical implementation. The pathological mechanisms of AD are remarkably complex, and relying on a change in a single biomarker concentration cannot achieve precise subtyping or early warning.

To address these challenges, different detection platforms have their own advantages and limitations. Colorimetric methods are easy to operate and do not require complex instruments, making them suitable for primary healthcare screening. The conventional colorimetric immunoassays such as ELISA are relatively simple to perform, but their sensitivity needs to be improved. Fluorescence methods have high sensitivity (up to the fg/mL level) and are suitable for diagnosis in memory clinics. Chemiluminescence has a sensitivity of pg/mL and high automation, making it suitable for detecting relatively abundant biomarkers. However, both fluorescence and chemiluminescence methods require relatively complex operation and high equipment cost. In addition, SPR, enabling label-free real-time detection, is suitable for therapeutic monitoring, and SERS, combining ultra-high sensitivity with multiplexing capability, is suitable for confirmatory and differential diagnosis in large medical centers. Nevertheless, these two methods involve complex chip/sample preparation procedures and low automation. Simoa can provide the highest sensitivity (fg/mL level) and meet the quantitative detection requirements of core AD blood biomarkers throughout the entire disease process.

The core challenges in the clinical application of optical methods with ultra-low sensitivity are the lack of systematic benchmarks against current clinical gold standards such as Aβ/Tau PET and mass spectrometry, the absence of standardized quality control systems, and the deficiency of multi-center, large-scale clinical validation data across different AD stages and subtypes. Blood protein biomarkers are suitable indicators for early screening, differential diagnosis, and progression monitoring of AD, but there is an urgent need to establish standardized testing protocols, quality control systems, and interpretation standards for detection results. It is also necessary to establish large-scale, multi-center prospective cohort studies to define reference intervals, diagnostic cutoffs, and clinical application pathways for AD protein biomarkers. These challenges do not mean that optical sensing platforms lack clinical potential. On the contrary, in recent years, an increasing number of studies have confirmed that optical sensing technologies using clinical serum or plasma samples can, in some cases, achieve diagnostic performance close to or even exceed traditional methods. Based on the above analysis, some innovative optical methods should not be viewed as independent alternatives to CSF or PET diagnosis, but rather as triage tools in the two-step workflow of “ triage-confirmation”. The positive results of optical methods should be further confirmed by gold standard methods to ensure diagnostic accuracy and reliability of diagnosis.

## 5. Conclusions

As the most common neurodegenerative disease worldwide, early diagnosis of AD remains a major challenge in clinical medicine. Novel methods relying on AD blood biomarkers, especially Aβ and Tau proteins along with their isoforms, exhibit significant advantages including easy sampling, minimal invasiveness, and good repeatability, thus representing a promising direction for early AD diagnosis. However, the extremely low concentrations of these biomarkers in blood impose stringent requirements on the sensitivity, specificity, and anti-interference capability of detection methods. Optical methods, characterized by high sensitivity, rapid response, and potential for miniaturization, provide new avenues to overcome these challenges. This review systematically summarizes recent advances in optical methods for the detection of Aβ and Tau proteins, including colorimetry, fluorescence, chemiluminescence, SPR, and SERS. Colorimetric strategies, including enzyme/nanozyme-catalyzed chromogenic reactions, aggregation-induced color changes, and lateral flow assays, have achieved rapid and intuitive detection. In fluorescence sensing, QDs and upconversion nanoprobes have been used as signal labels, and aptamer recognition and nucleic acid-based amplification techniques have significantly improved detection sensitivity. With polymers as the signal carriers, the sensitivity of chemiluminescence methods has been significantly enhanced. SPR techniques have achieved label-free and real-time detection of AD biomarkers in view of the high refractive index sensitivity of noble metal nanostructures. SERS with unique “molecular fingerprinting” ability and “hot-spot” enhancement effect shows exceptional advantages in label-free direct detection, aptamer/antibody-targeted recognition, and multi-biomarker analysis. In addition, some emerging integrated techniques such as SERS-coupled flow analysis, AI-assisted spectroscopic analysis, and single-molecule immunoassay show potential for diverse applications.

Despite significant progress in optical sensing of AD biomarkers, several challenges still need to be addressed to advance clinical translation. First, the complex pathology of AD requires the simultaneous detection of multiple biomarkers, but most of the current studies focus on the detection of a single analyte, limiting diagnostic accuracy. To overcome this, future efforts should prioritize the development of sensor arrays and multi-encoding strategies capable of multiplexed detection of Aβ42/Aβ40 ratio, p-Tau isoforms, and other biomarkers such as NfL and GFAP in a single assay platform. Second, the extremely low concentrations of AD biomarkers in blood samples, combined with severe matrix interference from abundant proteins and other biomolecules, demand further improvements in sensitivity, stability, and anti-fouling performance. The integration of single-molecule detection technologies and novel signal amplification strategies, together with the development of robust anti-interference interfaces, will be essential to enhance detection reliability in complex biological matrices. Third, most of the reported studies rely on standard addition methods with few serum samples for validation, and the lack of systematic evaluation using large, diverse clinical cohorts severely limits the clinical applicability and generalizability of current methods. Therefore, it is urgently needed to develop large-scale, multi-center prospective cohort studies to define reference intervals and diagnostic cutoffs across different populations. Fourth, the absence of standardized protocols for blood collection and storage, sensor fabrication, testing parameters, and data interpretation across different laboratories hinders the comparability and widespread adoption of experimental results. Integrating machine learning into the construction of standardized and transferable spectral databases and diagnostic models will provide a promising approach for intelligent and coordinated interpretation of results. Finally, the limited portability of current optical sensing platforms restricts their deployment in primary care and home-based settings. Developing intelligent portable detection devices, such as microfluidic chips, smartphone-based readers, and wearable sensors, will be critical to enable point-of-care testing and broaden access to early AD screening. In summary, optical biosensing technologies represent a promising avenue for early diagnosis of AD, but continuous efforts are still needed in terms of matrix interference mitigation, multiplexing capability, large-scale clinical validation, standardization, and device portability to promote their translation from laboratory research to clinical practice.

## Data Availability

Not applicable.
